# Vaccination generates broadly cross-neutralizing antibodies to the HIV Env apex

**DOI:** 10.1038/s41586-026-10429-3

**Published:** 2026-04-29

**Authors:** Javier Guenaga, Monika Ádori, Shridhar Bale, Swastik Phulera, Ioannis Zygouras, Fabian-Alexander Schleich, Xaquin Castro Dopico, Sashank Agrawal, Miyo Ota, Richard Wilson, Jocelyn Cluff, Tamar Dzvelaia, Marco Mandolesi, Wen-Hsin Lee, Agnes A. Walsh, Mariane B. Melo, Laurent Verkoczy, Darrell J. Irvine, Martin Corcoran, Ian A. Wilson, Diane Carnathan, Guido Silvestri, Andrew B. Ward, Gabriel Ozorowski, Gunilla B. Karlsson Hedestam, Richard T. Wyatt

**Affiliations:** 1 Department of Immunology and Microbiology, The Scripps Research Institute, La Jolla, CA USA; 2 Department of Microbiology, Tumor and Cell Biology, Karolinska Institutet, Stockholm, Sweden; 3 Department of Integrative Structural and Computational Biology, The Scripps Research Institute, La Jolla, CA USA; 4 Applied Biomedical Science Institute, San Diego, CA USA; 5 Howard Hughes Medical Institute, Chevy Chase, MD USA; 6 Division of Microbiology and Immunology, Emory National Primate Research Center, Emory University, Atlanta, GA USA; 7 Science for Life Laboratory, Stockholm, Sweden

**Keywords:** Protein vaccines, HIV infections

## Abstract

As a chronically replicating virus, HIV has evolved extreme sequence variability and effective shielding of functionally constrained spike protein determinants by host-derived glycans^1^. Broadly neutralizing antibodies, although rare, can be isolated from people living with HIV, revealing conserved envelope glycoprotein (Env) sites as key targets for vaccine development^2–4^. One such target is the apex of the Env spike. Here we identify a vaccination strategy using heterologous HIV Env trimers covalently coupled to liposomes for multivalent display that resulted in the elicitation of cross-neutralizing HIV serum antibody responses in all trimer-liposome-immunized non-human primates. Critically, we isolated monoclonal antibodies from multiple macaques that cross-neutralize divergent HIV clinical isolates. High-resolution cryogenic electron microscopy structural analyses of monoclonal antibodies from four different macaques demonstrate that they target the Env trimer apex in a manner highly similar to that of the human-infection-elicited, apex-directed broadly neutralizing antibody PG9, representing a substantial advance in HIV vaccine development.

## Abstract (web summary)

A vaccination strategy using heterologous HIV Env trimers covalently coupled to liposomes for multivalent display generates cross-neutralizing HIV serum antibody responses in non-human primates, mimicking infection-elicited apex-targeting broadly neutralizing antibodies.

## Main

The development of an effective prophylactic HIV vaccine capable of reducing the spread of the virus remains a high-priority public health goal. However, chronic HIV replication in infected hosts drives immune evasion^5–7^, making vaccine development extremely challenging. The sole neutralizing determinant on the surface of the virus is the trimeric envelope glycoprotein (Env) spike, which has evolved to evade host antibody responses by incorporating host-derived N-glycans and altering variable domains during replication^1,8^. Broadly neutralizing antibodies (bNAbs), which arise infrequently in people living with HIV-1, can penetrate and, in some cases, co-recognize components of the glycan shield and underlying protein structures, thereby revealing sites of vulnerability. These include the Env trimer apex, the primary CD4 receptor binding site, V3 glycan site and functionally constrained regions of the HIV spike proximal to the viral membrane^9,10^.

Elicitation of apex-targeting bNAbs is of particular interest for vaccine design, as such antibodies often require lower levels of somatic hypermutation than other HIV bNAbs to gain broad neutralizing activity^11^. Several potent apex-directed bNAbs have been isolated from people living with HIV-1^3,12–17^. Generally, the HIV-induced apex bNAbs display a very long heavy-chain-complementarity-determining region 3 (HCDR3) characterized by negatively charged tips that interact with the basic HIV Env variable region 2 (V2) C-strand, such as those found in PG9/16, PGT145, VRC26 and CH01-4^18–22^. The glycan-shrouded apex of the Env spike is recognized by the bNAb HCDR3s that penetrate the glycan shield interacting with both the surrounding glycans and the semi-conserved basic C-strand of V2^11,21–24^. The maturation of these antibodies is driven by the co-evolution of the virus and antibody interactions that include both glycan and protein contacts^23,25^.

Studies show that infection with chimeric simian–human immunodeficiency viruses (SHIVs) elicits apex-targeting bNAbs in non-human primates (NHPs), such as RHA1^26^, as well as other apex-directed cross-neutralizing monoclonal antibodies (mAbs)^27^. These findings validate macaques as a suitable model for evaluation of vaccines aimed to stimulate such responses^26^. The SHIV-induced cross-neutralizing serum activity detected in the RHA1-generating NHP was informative for identifying virus trimers capable of eliciting apex-directed antibody responses (that is, Q23.17 and ZM233 were potently neutralized). Moreover, SHIV-infection-induced apex-targeting bNAbs can inform HIV vaccine design. However, B cell activation and general immune stimulus by a replicating virus differ substantially from that induced by recombinant protein subunit vaccines. It is less certain whether subunit vaccination can elicit comparable apex-targeting neutralizing antibody responses in animal models, an important first step towards translatable human vaccine strategies.

Here we screened a set of native-like HIV Env trimers using the apex-targeting bNAb, RHA1 and its inferred germline (IgL) reverted variant (IgL RHA1) to identify a potential priming immunogen. We selected Q23.17-HIV-strain-derived native flexibly linked (NFL)-stabilized trimers based on their avid binding to the mature RHA1 bNAb and detectable binding to the IgL RHA1 mAb^28–30^. To enhance B cell priming, we covalently arrayed the Q23 trimers on synthetic liposomes (Q23 trimer-liposomes)^31^ and compared the liposome-arrayed platform with soluble NFL trimers in rhesus macaques. After Q23 NFL priming and sequential Env boosting with the heterologous ZM233 NFL trimers, we detected robust serum neutralization of both Q23.17 and ZM233M.6 pseudoviruses in animals from both groups of NHPs. Apex-directed electron microscopy-based polyclonal epitope mapping (EMPEM) densities were detected in all NHPs immunized with the trimer liposomal array. After sequential boosting with two additional stabilized trimers (WITO and 0014824 NFLs), we measured tier-two cross-neutralization in the sera of all trimer-liposome immunized NHPs. On the basis of these encouraging results, we isolated a panel of 58 Env-binding mAbs and characterized their genetic, functional and structural properties. Cryogenic electron microscopy (cryo-EM) structures of mAb–trimer complexes at a high resolution showed that mAb apex targeting mirrors the binding mode of the infection-elicited human bNAb PG9. These findings demonstrate that high-density liposome arrays of native-like Env trimers effectively activate apex-targeting B cells, eliciting antibody responses that recapitulate features described in human infection-driven bNAbs.

## Env binding by a germline-reverted bNAb

**Fig. 1:**
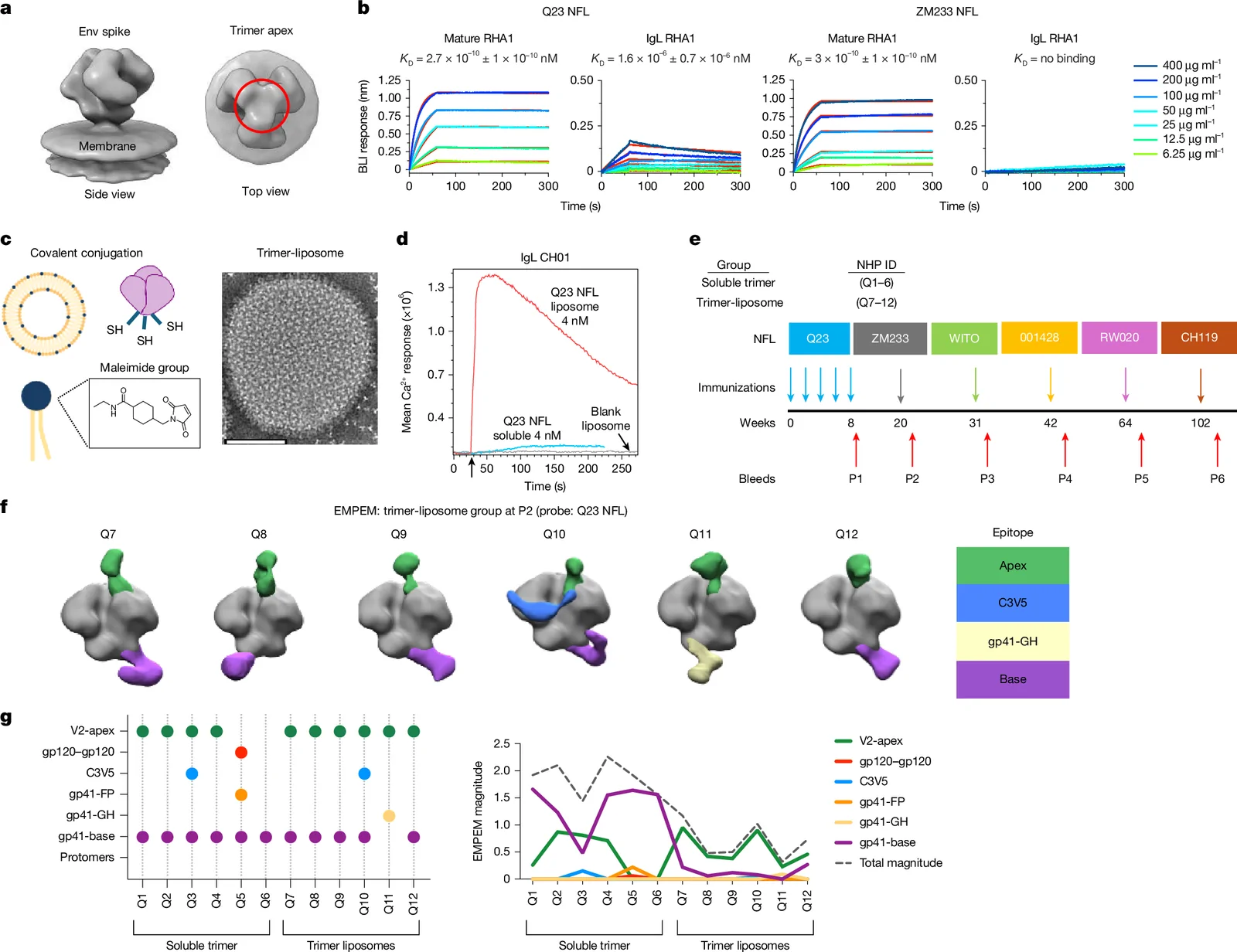
Immunization with Env NFL trimer-liposomes elicits apex-directed, cross-neutralizing serum antibodies in all NHPs. **a**, Surface representation of the HIV-1 Env trimer on the membrane (left) and trimer apex top view, adapted from its simian variant spike (EMDB: EMD-5273) (right). **b**, BLI binding analysis of Q23 and ZM233 NFL trimers to mature and inferred germ-line reverted RHA1 mAbs (IgL RHA1). **c**, Diagram of liposome conjugation to trimers (left). Right, negative-stain EM image of trimer-liposomes. Scale bar, 100 nm. The diagram was created using BioRender; Bale, S. https://BioRender.com/bww3j5e (2026). **d**, Calcium flux binding response of IgL CH01 B cells to Q23 trimer-liposomes or soluble trimer. **e**, Immunization regimen and NFL trimers used as immunogens. **f**, EMPEM analysis of NHPs Q7–Q12 after immunization with Q23 and ZM233 NFL trimers (the P2 bleed timepoint). **g**, The per-epitope (solid lines) and total (dashed line; sum of all per-epitope magnitudes) EMPEM magnitude by animal. The EMPEM magnitude is the average number of antibodies bound per trimer. The V2-apex magnitude is normalized by 3× to account for a 1:1 Fab:trimer binding stoichiometry. BLI and calcium flux experiments were repeated twice with similar results. The negative-stain EM image of trimer-liposomes was selected as a representative of several imaging sessions. Source data

**Extended Data Fig. 1:**
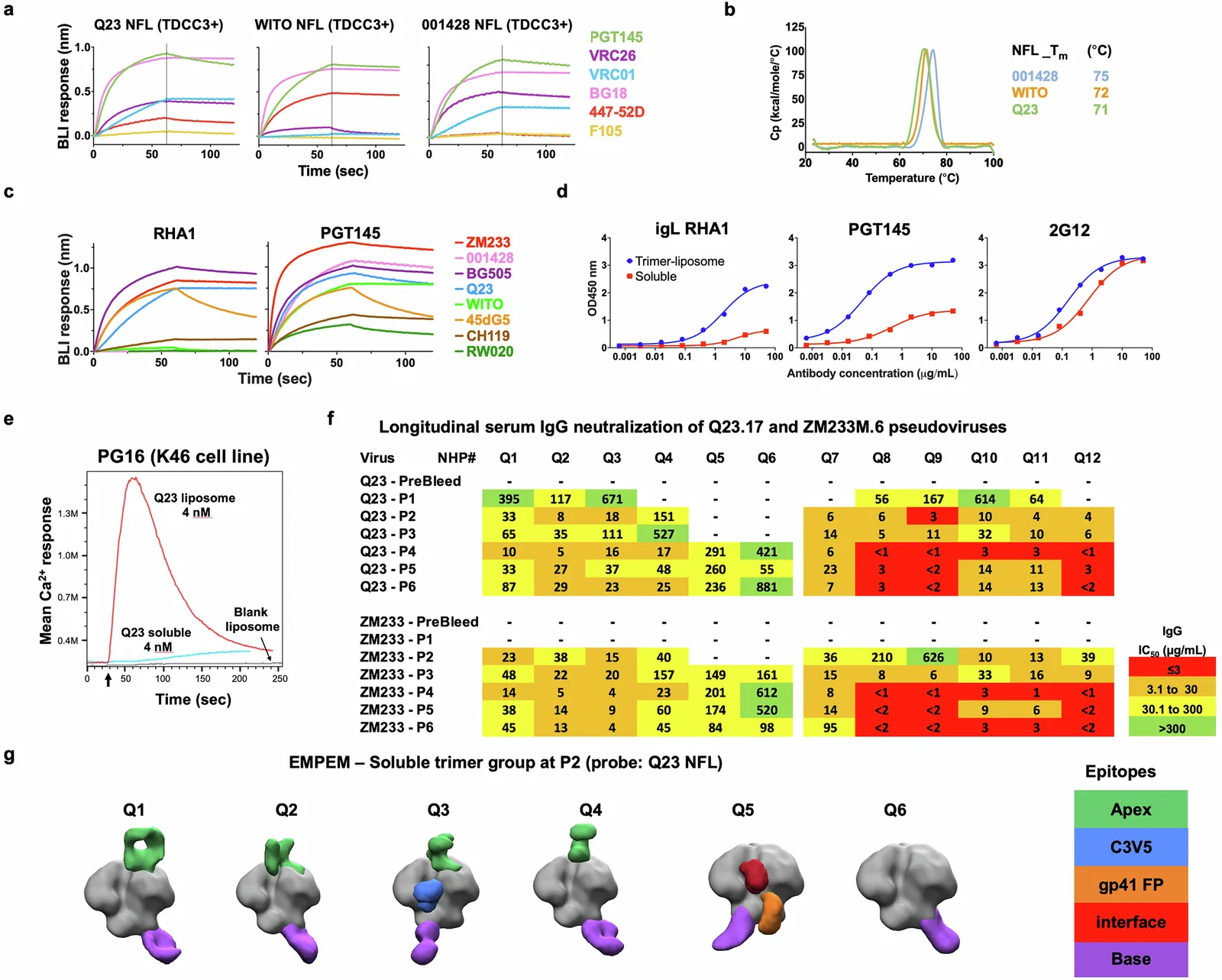
Biochemical analysis of NFL trimers, trimer-liposomes, serum neutralization and EMPEM analysis. (**a**) BLI binding of NFL trimers to representative antibodies. (**b**) DSC of NFL trimers. (**c**) BLI binding of RHA1 and PGT145 to NFL trimers. (**d**) ELISA comparing the binding of Q23 NFL trimer-liposomes and soluble Q23 trimers to representative antibodies. (**e**) Calcium flux analysis of PG16 expressing K46 cells. (**f**) Longitudinal IgG neutralization titres (IC_50_ μg/mL) against HIV-1 Q23.17 and ZM233M.6 pseudoviruses. (**g**) EMPEM analysis of Q1-Q6 NHPs at P2. BLI, DSC, Calcium Flux, ELISA and neutralization assays were repeated twice with similar results. Source data

The apex of the HIV Env spike is often targeted by infection-derived bNAbs and represents a vaccine target (Fig. 1a). It was previously demonstrated that CH505 SHIV infection generated apex-directed polyclonal responses that potently neutralized Q23.17, ZM233M.6 and WITO.33 HIV-1 strains, which are highly sensitive to known infection-elicited V2-apex bNAbs^24,26^. Serum cross-neutralizing activity was detected within a year after SHIV infection and RHA1, an apex-targeting bNAb, was isolated from one of the NHPs. On the basis of these findings, we generated soluble, stabilized, near-native NFL trimers from several of these neutralization-sensitive strains, and confirmed their conformational integrity, as described here and previously using biolayer interferometry (BLI) and differential scanning calorimetry (DSC)^28–30,32^ (Extended Data Fig. 1a–c). The NFL trimers were tested for recognition by the apex-directed NHP bNAb RHA1 and the human PGT145 (Extended Data Fig. 1c). We measured nanomolar binding affinity of mature RHA1 to NFL trimers derived from the Q23.17 and ZM233M.6 HIV-1 strains, while only the Q23 NFL trimer displayed micromolar binding affinity to the germline-reverted antibody IgL RHA1 (Fig. 1b). The germline-reverted RHA1 mAb was designed by reverting both light (LC) and heavy (HC) chain residues to their assigned germline sequences, retaining only the mature HCDR3^33^. We next evaluated whether high-avidity, multivalent Q23 NFL trimers arrayed covalently on liposomes (Fig. 1c) would enhance binding to IgL RHA1 as determined by enzyme-linked immunosorbent assay (ELISA). Compared with soluble trimers, liposome-arrayed Q23 NFL trimers displayed stronger binding to the IgL RHA1 and to the mature PGT145. Comparable binding was detected by the glycan-dependent bNAb 2G12, confirming that equivalent levels of trimers were captured (Extended Data Fig. 1d). To assess B cell activation by the NFL trimer liposomal array, we measured calcium flux in primary mouse B cells expressing the apex-targeting IgL variant of the human bNAb, CH01, as well as in the mouse K46 cell line expressing the mature bNAb PG16. We detected a substantial increase in intracellular Ca^2+^ flux induced by the multivalent Q23 trimer-liposomes compared with the flux induced by the soluble trimers (Fig. 1d and Extended Data Fig. 1e), indicating that the multivalent Q23 trimer array could potentially activate naive B cells more effectively.

## Apex cross-neutralizing serum antibodies

Twelve NHPs were immunized with Q23 NFL trimers, administered either as soluble trimers (Q1–Q6) or covalently coupled to high-valency array as trimer-liposomes (Q7–Q12) (Fig. 1e). The SMNP adjuvant was used throughout^34^. To increase engagement of the relatively infrequent apex-bNAb precursors in the NHP repertoire, we opted for a fractionated Q23 NFL priming, dosed equivalently over 8 weeks. Serum-purified IgG collected after the Q23 and ZM233 NFL sequential immunization (hereafter, the P2 timepoint) showed autologous neutralization of both Q23.17 and ZM233M.6 pseudoviruses in four NHPs from the soluble group and in all six NHPs from the trimer-liposome group (Extended Data Fig. 1f). The serum-purified IgG was concentrated to a level equivalent to that naturally found in the serum (approximately 10 mg ml^−1^) and neutralization was reported as 50% inhibitory concentrations (IC_50,_ μg ml^−1^). All NHPs in both groups developed and intensified Q23.17 and ZM233M.6 serum neutralizing antibody responses over subsequent immunizations, administered every 12 weeks using NFL Env trimers from heterologous HIV strains (WITO.33, 001428-2.42, RW020.2 and CH119.10; timepoints P3–P6, denoting bleeds collected two weeks after the third, fourth, fifth and sixth NFL trimer vaccinations) indicating effective cross-boosting (Extended Data Fig. 1f). To determine whether the antibody responses elicited after the second trimer immunization with the ZM233 NFL targeted the HIV Env apex, we performed EMPEM to visualize serum antibodies in a complex with the NFL trimer immunogen (test bleed at the P2 timepoint). EMPEM analysis of the serum-IgG-derived antigen-binding (Fab) fragments in a complex with the Q23 NFL trimer revealed binding densities at the trimer apex in all NHPs from the trimer-liposome group (Fig. 1f,g) and in four NHPs from the soluble group (Fig. 1g and Extended Data Fig. 1g). Off-target base responses were detected in most NHPs; however, EMPEM magnitude analysis showed that the base responses were substantially lower in the trimer-liposome samples (Fig. 1g).

**Extended Data Fig. 2:**
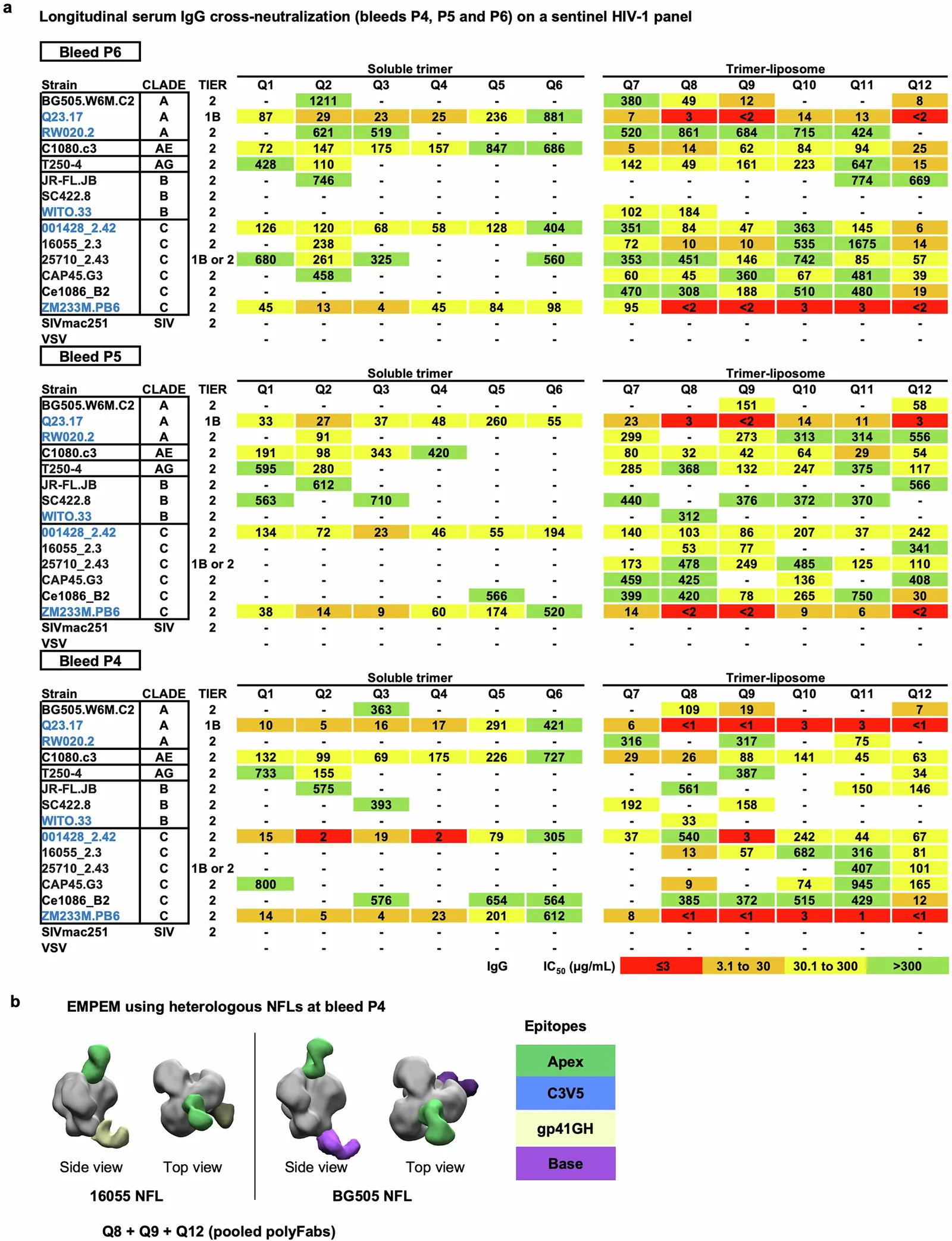
Longitudinal neutralization titres of serum IgG against a sentinel panel. (**a**) Serum IgG neutralization IC_50_ titres (µg/mL) at P4, P5, and P6 corresponding to bleeds collected after the fourth, fifth, and sixth NFL immunization. Blue font denotes viruses whose Env sequences are represented in the NFL vaccines while black font denotes heterologous viruses. (**b**) EMPEM analysis of pooled sera from NHPs Q8, Q9, and Q12 at P4. With antibodies coloured according to the key in Extended Data Fig. 1g. Neutralization assays were repeated twice with similar results.

After immunization with the fourth Env 001428 NFL trimer, the IgG samples (test bleed at the P4 timepoint) from most animals vaccinated with trimer-liposomes demonstrated superior neutralizing activity against a sentinel virus panel compared with those from the soluble trimer group (broader and more potent). This superiority persisted after immunizations with RW020 and CH119 NFL trimers (test bleeds at the P5 and P6 timepoints) (Extended Data Fig. 2a). Based on these data, we analysed the serum antibodies using EMPEM to define the predominant cross-binding specificities after four trimer immunizations. We selected three NHPs from the trimer-liposome group (Q8, Q9 and Q12) that displayed neutralization activity against several heterologous HIV strains, including 16055-2.3 and BG505.W6M.C2. Notably, NFLs derived from these strains were not part of the immunization regimen, providing a filter to analyse cross-binding with the potential to indicate cross-neutralizing specificity. We therefore analysed the pooled sera of these animals using EMPEM against the heterologous trimers and detected polyclonal antigen-binding-fragment binding densities targeting the Env apex in both 16055 and BG505 NFL trimer–Fab complexes. These data strongly suggested that the cross-neutralization was probably mediated by the serum IgG targeting the Env apex as indicated by the EMPEM polyclonal antigen-binding-fragment densities (Extended Data Fig. 2b).

**Fig. 2:**
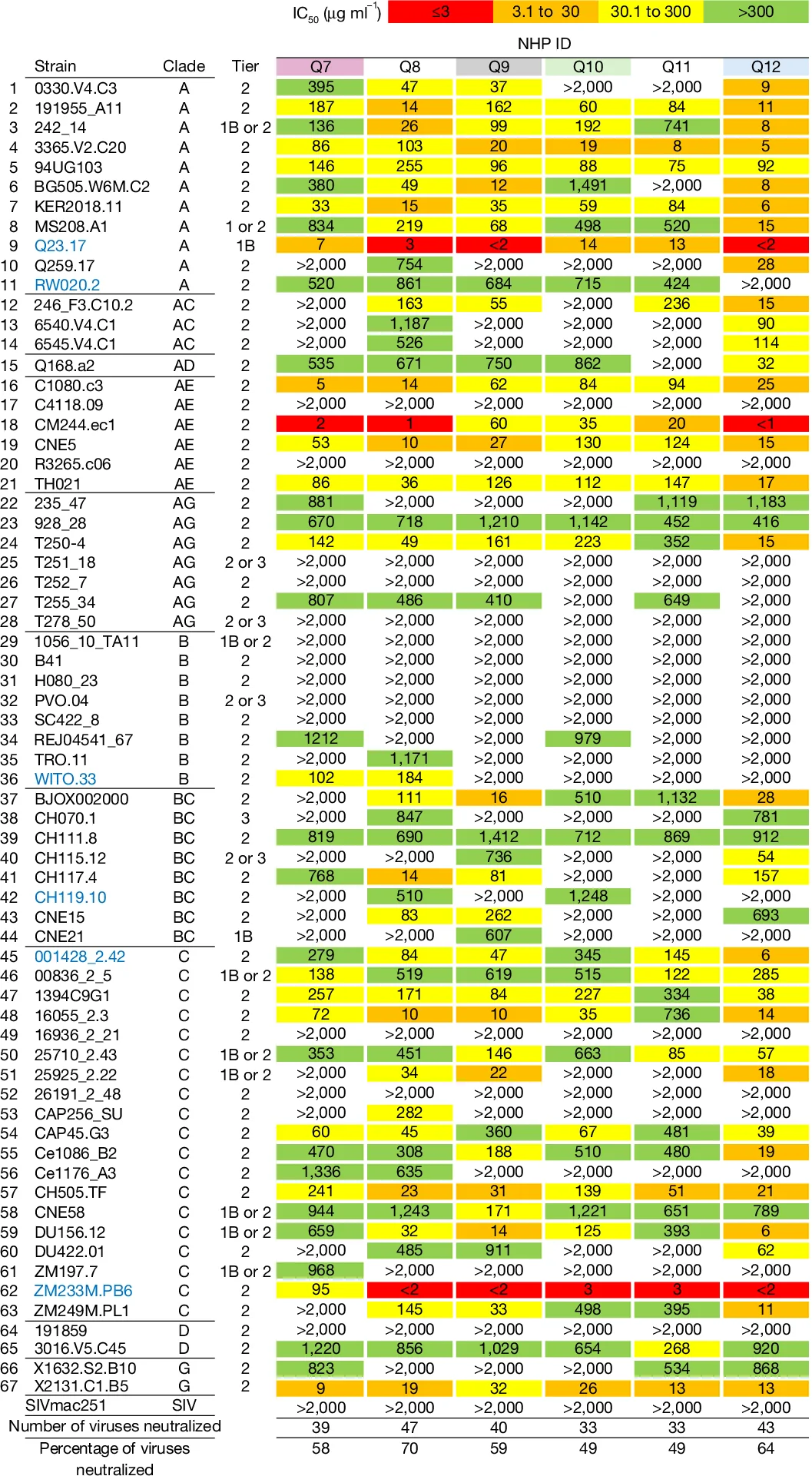
Neutralizing titres against a 67-virus panel of clinical HIV-1 isolates. Total serum IgG IC_50_ neutralization titres (µg ml^−1^) of bleed samples of Q7–Q12 NHPs at timepoint P6, after immunization with six NFL trimers. Blue font denotes viruses of which the Env sequences are represented in the NFL vaccines (autologous); black font denotes heterologous viruses. Neutralization assays were repeated twice with similar results.

Additional sequential heterologous trimer boosting with RW020 and CH119 NFL trimer-liposomes (P5 and P6 timepoints) generally broadened the cross-neutralizing activity. Specifically, test bleed samples of NHPs Q7, Q8, Q9, Q10 and Q11 at the P6 timepoint gained neutralization activity (1–6 viruses) compared with the P4 timepoint against this sentinel viral panel (Extended Data Fig. 2a). We next analysed the P6 serum IgG samples from the trimer-liposome group against a larger virus panel containing a set of diverse HIV-1 strains from multiple clades. Overall, serum IgG from all six NHPs immunized with the trimer-liposomes neutralized more than 49% of viruses in the 67-virus panel with a range of potencies. Notably, NHPs Q8 and Q12 neutralized 70% and 64% of the viruses, respectively (Fig. 2). From these data, we conclude that our vaccine strategy, prime–boosting with heterologous NFL trimers covalently arrayed on liposomes, elicited apex-directed, cross-neutralizing antibodies in all NHPs after four Env inoculations, and that the fifth and sixth inoculations further broadened the cross-neutralizing activity.

## Neutralization by vaccine-elicited mAbs

**Fig. 3:**
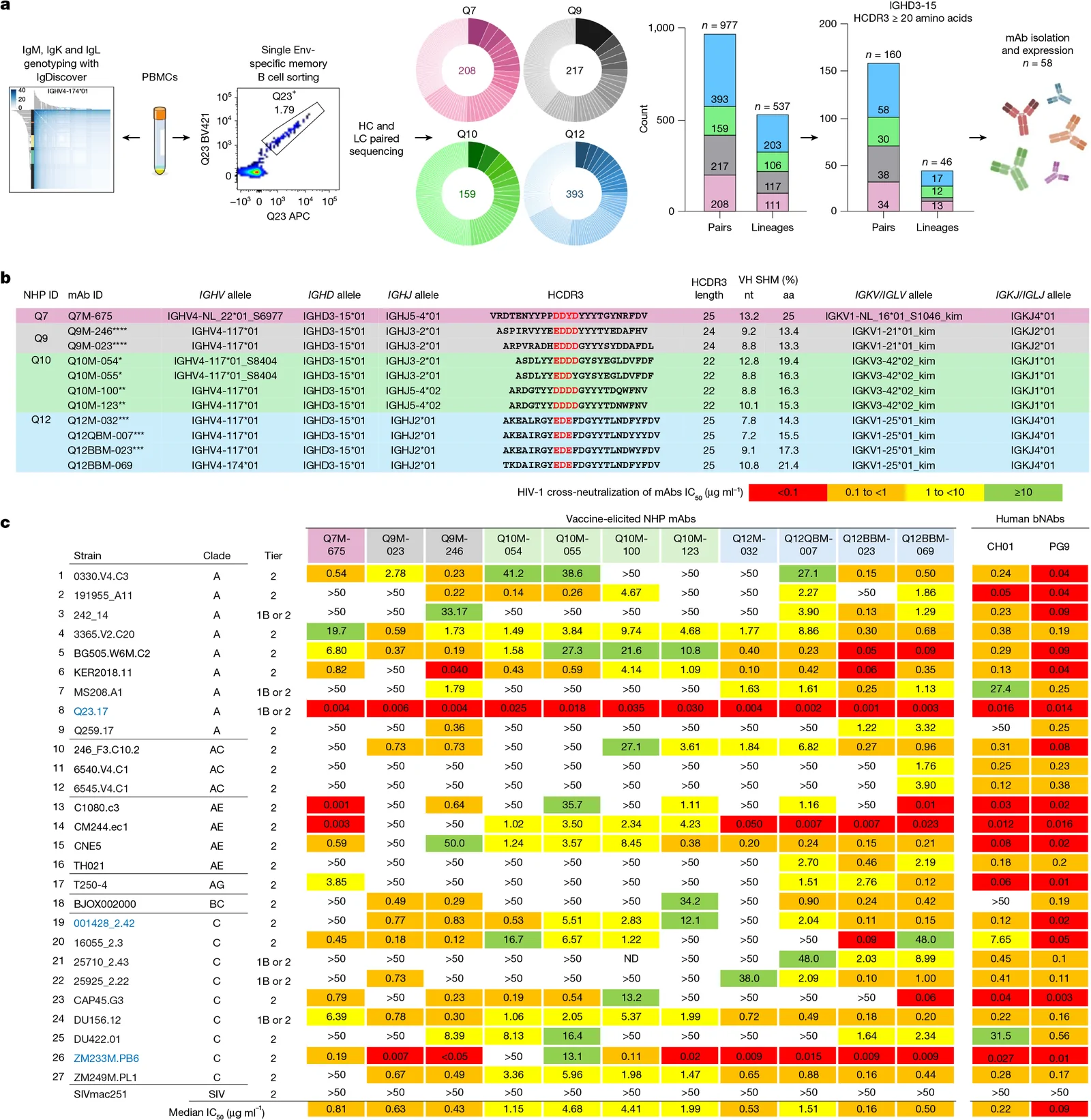
Env-specific memory B cell sorting, sequence analysis and neutralization of clinical isolates by selected mAbs. **a**, Overview of IgG genotyping, Env-specific single memory B cell sorting, analysis of paired HC and LC sequences, and expression of mAbs. **b**, Genetic properties of mAbs that neutralize more than four HIV-1 strains in the initial panel (clonally related mAbs are indicated with asterisks, where mAbs with the same number of asterisks belong to the same lineage). *IGHD3-15* gene central acidic residues are highlighted in red. **c**, Neutralizing inhibitory concentrations IC_50_ (µg ml^−1^) for selected mAbs against a subpanel of 27 HIV-1 clinical isolates. The human bNAbs CH01 and PG9 are included for comparison. Neutralization assays were repeated twice with similar results. Source data

To define the antibody reactivities responsible for the serum IgG cross-neutralization detected in the trimer-liposome-immunized NHPs, we isolated single Env-binding memory B cells from peripheral blood mononuclear cells (PBMCs) collected at timepoints P4, P5 and P6, using Q23, BG505 and 16055 NFL trimers as probes for flow-cytometry-based sorting (Fig. 3a, Supplementary Fig. 1 and Supplementary Table 1a). Guided by the serum neutralization data, we focused our analysis on NHPs Q7, Q9, Q10 and Q12 from which we isolated 208, 217, 159 and 393 paired HC and LC sequences, respectively. To enable precise allele assignments and definition of antibody clonal relationships, we genotyped the NHPs for their immunoglobulin allele content using IgDiscover and we assigned all HCs and LCs to the closest germline alleles present in that animal^35–37^. Using the IgDiscover clonotypes module, the antibodies from NHPs Q7, Q9, Q10 and Q12 were grouped into 111, 117, 106 and 203 clonal lineages, respectively (Fig. 3a (left)). Clonal lineages were defined as groups of sequences sharing the same heavy and light chain V and J gene alleles, with identical CDR3 lengths and a minimum of 80% nucleotide identity in the HCDR3. To identify antibodies with features characteristic of V2-apex bNAbs, we selected those with HCDR3s of at least 20 amino acid length and using the *IGHD3-15* gene (Supplementary Table 1b). The core of the macaque *IGHD3-15* gene contains a negatively charged motif (Fig. 3b) that can interact with the semi-conserved C-strand of Env V2 (residues 164–172), a region that is often basic in overall charge^24^. These HCDR3 features, also found in RHA1, are characteristic of apex-targeting bNAbs, including those isolated from SHIV-infected NHPs^26,27^. Applying these criteria resulted in a total of 160 HC–LC pairs, 34 from Q7, 38 from Q9, 30 from Q10, and 58 from Q12, grouped into 13, 4, 12 and 17 clonal lineages, respectively (Fig. 3a (right)).

**Extended Data Fig. 3:**
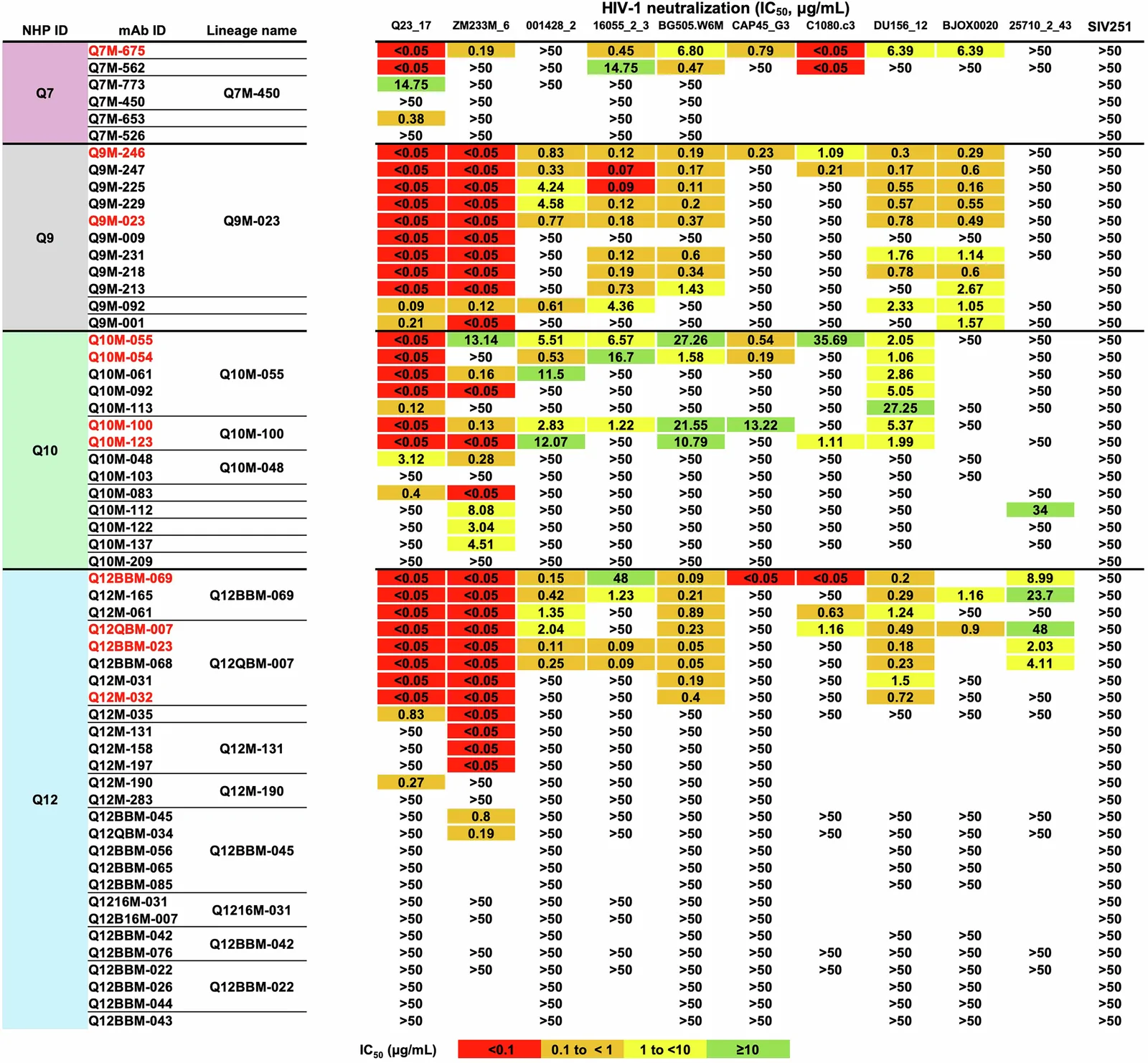
Neutralization titres of mAbs against a sentinel viral panel. Initial screening of expressed macaque mAbs cloned from NHPs Q7, Q9, Q10, and Q12 against a small panel of viruses.

From the 160 HC–LC pairs with V2-apex features from NHPs Q7, Q9, Q10 and Q12, we cloned 58 pairs, expressed them as mAbs and assessed their neutralizing activity on a sentinel panel of HIV-1 strains. This initial panel incorporated viruses from multiple clades that are sensitive to neutralization by human-infection-elicited apex-directed bNAbs, such as PG9/16, PGT145, PGDM1400 and VRC26^3,14,16,17^. In a few cases, these isolates are also neutralized by the germline-reverted or unmutated common ancestor (UCA) variants of the mature apex-directed bNAbs^24,38^. Another characteristic of these isolates is that they lack a glycan at the Env position Asn130, proximal to the Asn160 glycan on the trimer cap, probably increasing bNAb accessibility to the apex epitopes. Out of 347 viruses (the 208 panel plus the Seaman panel) used in a previous report^39^, about 50% of the isolates lack this Asn130 glycan. Here we found that several of the signature-defined mAbs isolated from NHPs Q7, Q9, Q10 and Q12 displayed potent autologous neutralization of the pseudoviruses Q23.17 and ZM233M.6, as well as heterologous cross-neutralization of an initial panel of cross-clade HIV-1 clinical isolates (Extended Data Fig. 3).

**Extended Data Fig. 4:**
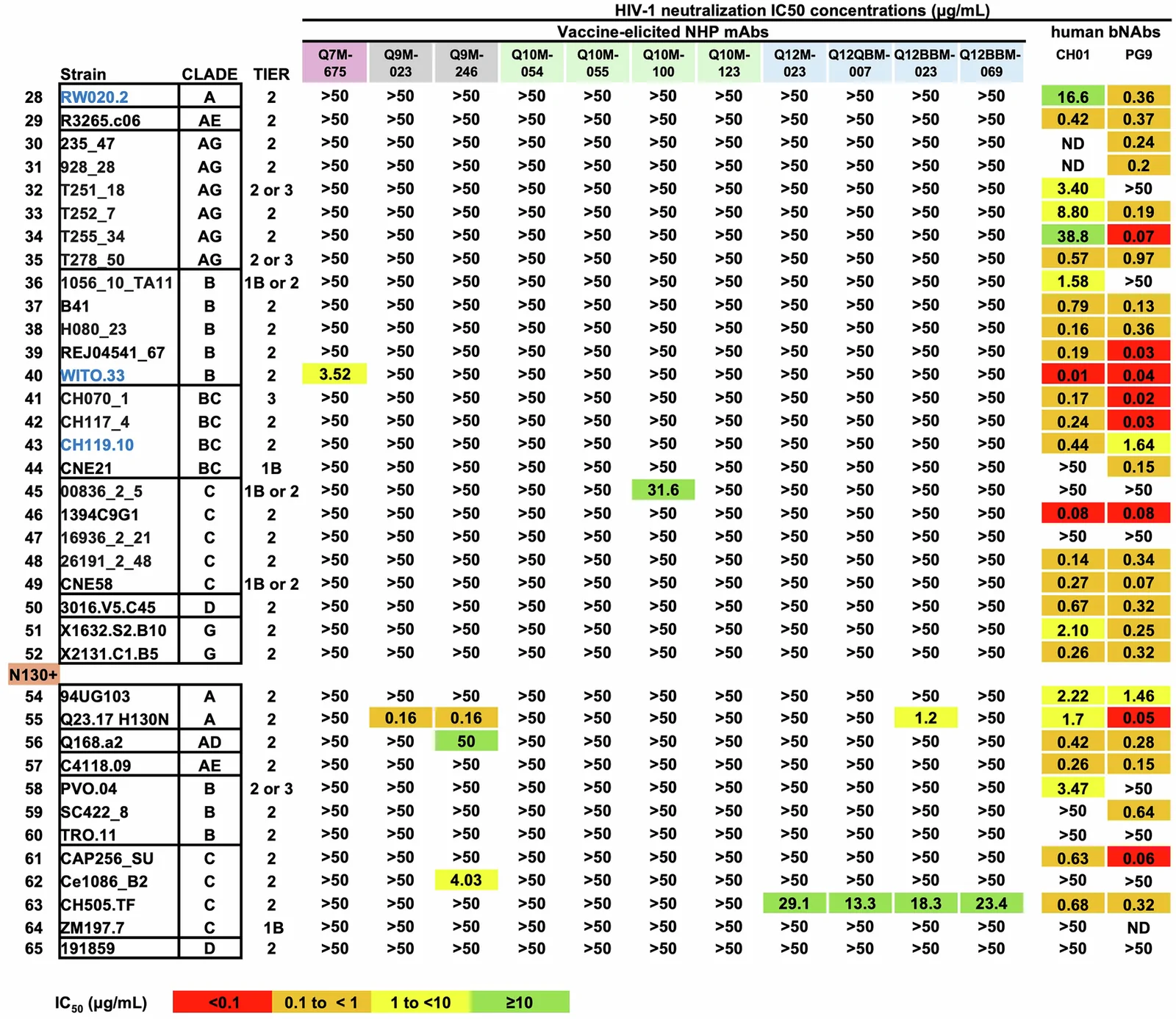
Neutralization titres of selected mAbs in an expanded viral panel. Macaque mAbs were tested against additional viruses lacking N130 glycan (28–52) and viruses with N130 glycan (54–65). Neutralization assays were repeated twice with similar results.

We next selected the 11 broadest vaccine-elicited mAbs isolated from NHPs Q7, Q9, Q10 and Q12, corresponding to 6 distinct clonal lineages (Fig. 3b and Supplementary Table 1b) and assessed them on a larger panel of 52 Asn130-lacking viruses (Fig. 3c and Extended Data Fig. 4). We included the infection-elicited apex bNAbs CH01 and PG9 as comparative controls in this analysis. The mAbs from NHP Q12 were the most broad and potent—particularly Q12BBM-069, Q12QBM-007 and Q12BBM-023, which neutralized 27, 21 and 22 isolates with median IC_50_ values of 0.50 µg ml^−1^, 1.51 µg ml^−1^ and 0.16 µg ml^−1^, respectively (Fig. 3c). Moreover, we tested 12 Asn130 glycan possessing viruses, as well as an engineered Q23.17 variant (H130N) in which the potential N-linked glycosylation site was modified to introduce the naturally lacking Asn130 glycan (Extended Data Fig. 4). Note that Q9M-246 neutralized three Asn130-bearing isolates, suggesting that some of the vaccine-elicited antibodies in the study can overcome the potential Asn130 clash. Moreover, the Q12 mAbs weakly neutralized the Asn130 glycan-containing CH505 T/F clinical isolate but did not neutralize other isolates tested here that naturally contain this apex N-glycan.

## Env apex targeting revealed by cryo-EM

**Fig. 4:**
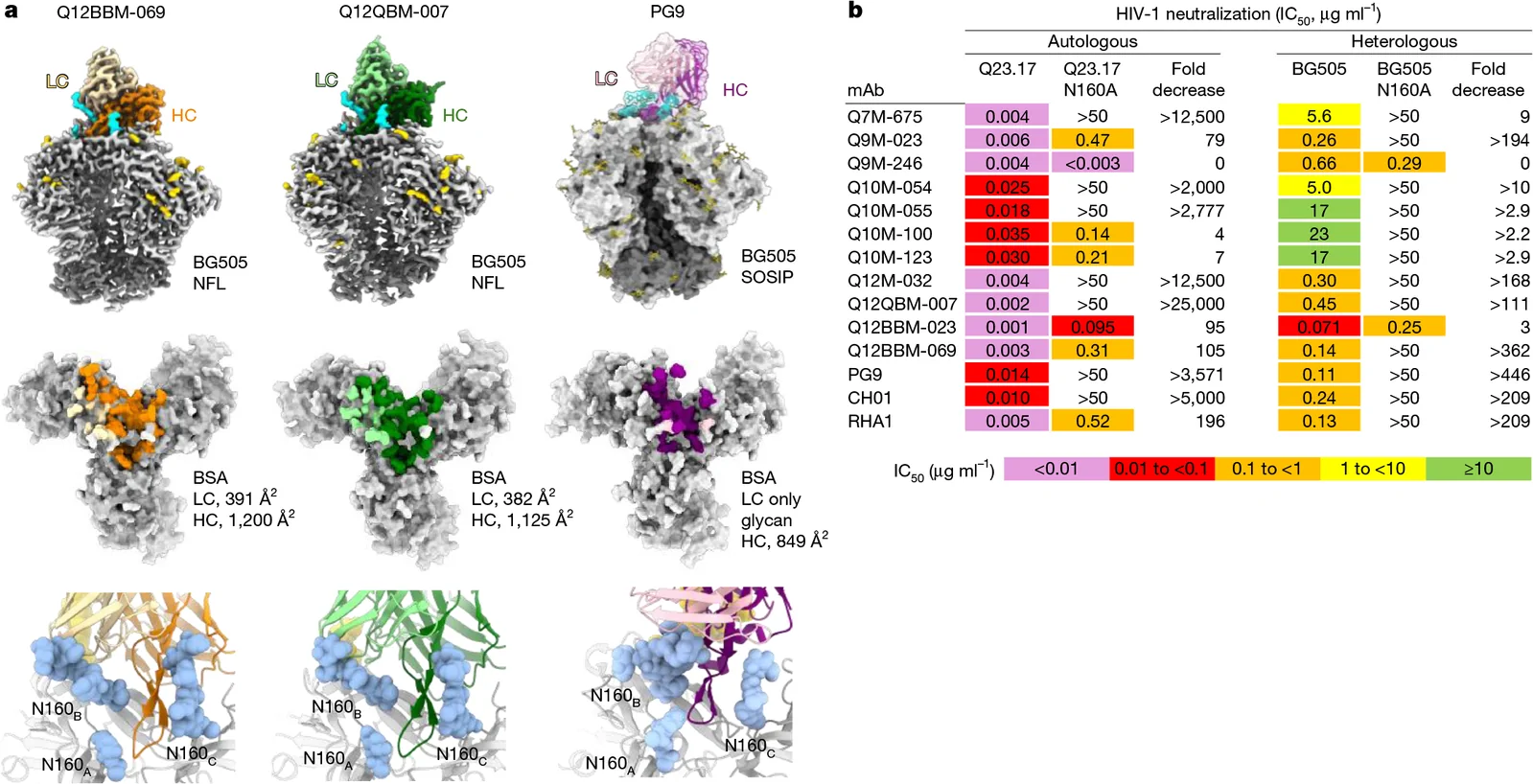
Cryo-EM structures of mAbs from NHP Q12 reveal binding at the Env trimer-apex. **a**, Cryo-EM maps and models of Q12BBM-069 and Q12QBM-007 Fabs in a complex with BG505 NFL compared with a cryo-EM model of the bNAb PG9 bound to ApexGT3.N130 BG505 SOSIP trimer (PDB: 7T77) (top). Middle, epitope footprint of mAbs on the surface of Env with buried surface area measurements. Bottom, interaction of the HCDR3 loops with the Asn160 glycans. **b**, Neutralizing titres of mAbs against HIV-1 Q23.17 and BG505 with and without the Asn160 glycan. Neutralization assays were repeated twice with similar results.

**Extended Data Fig. 5:**
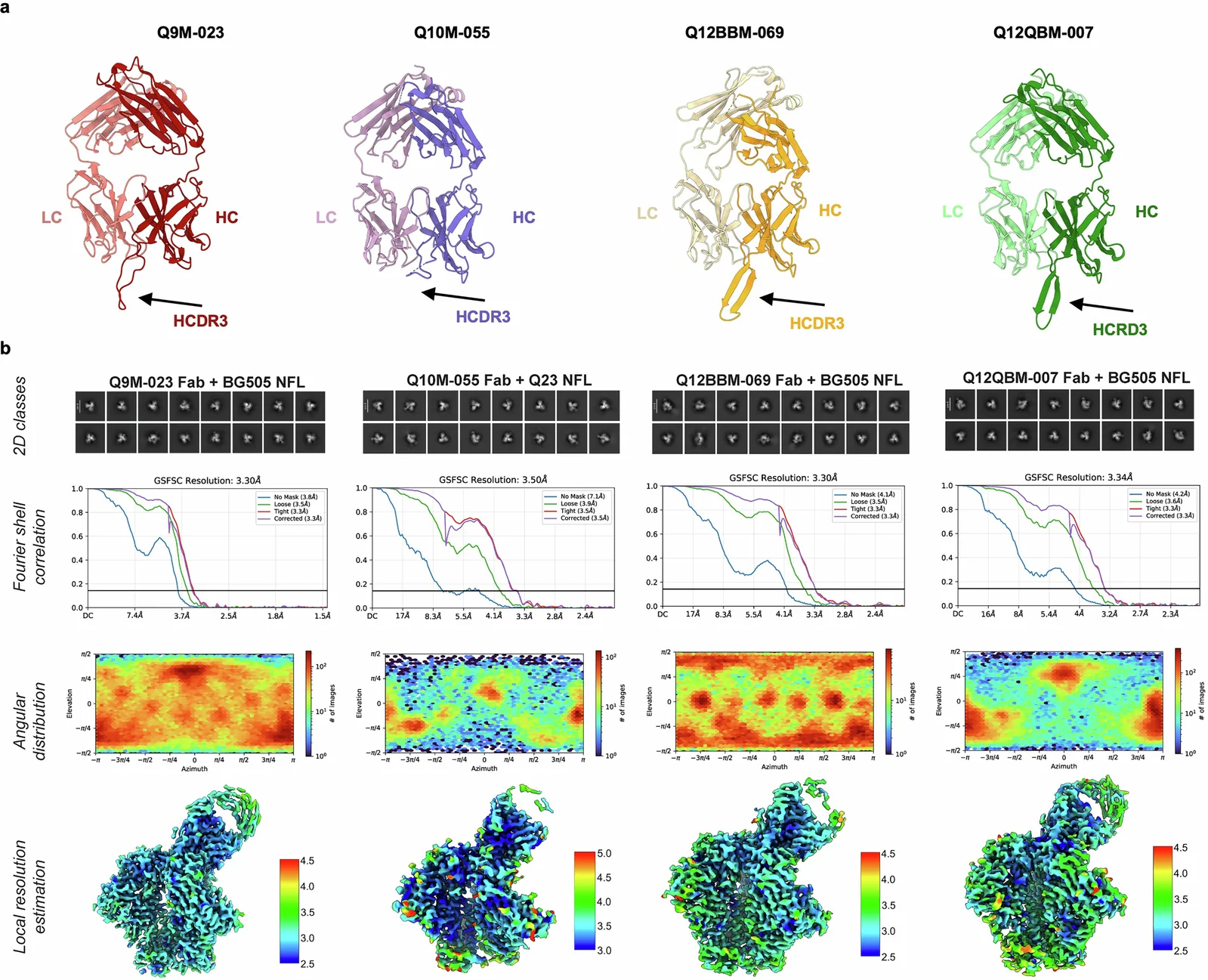
Crystal structures of Fabs and CryoEM reconstruction statistics. (**a**) Crystal structures of unliganded Fabs. HCDR3 is labelled. (**b**) Representative 2D class averages, Fourier shell correlation resolution estimate, angular distribution of observations, and local resolution estimation (in Å) of the cryoEM datasets.

**Extended Data Fig. 6:**
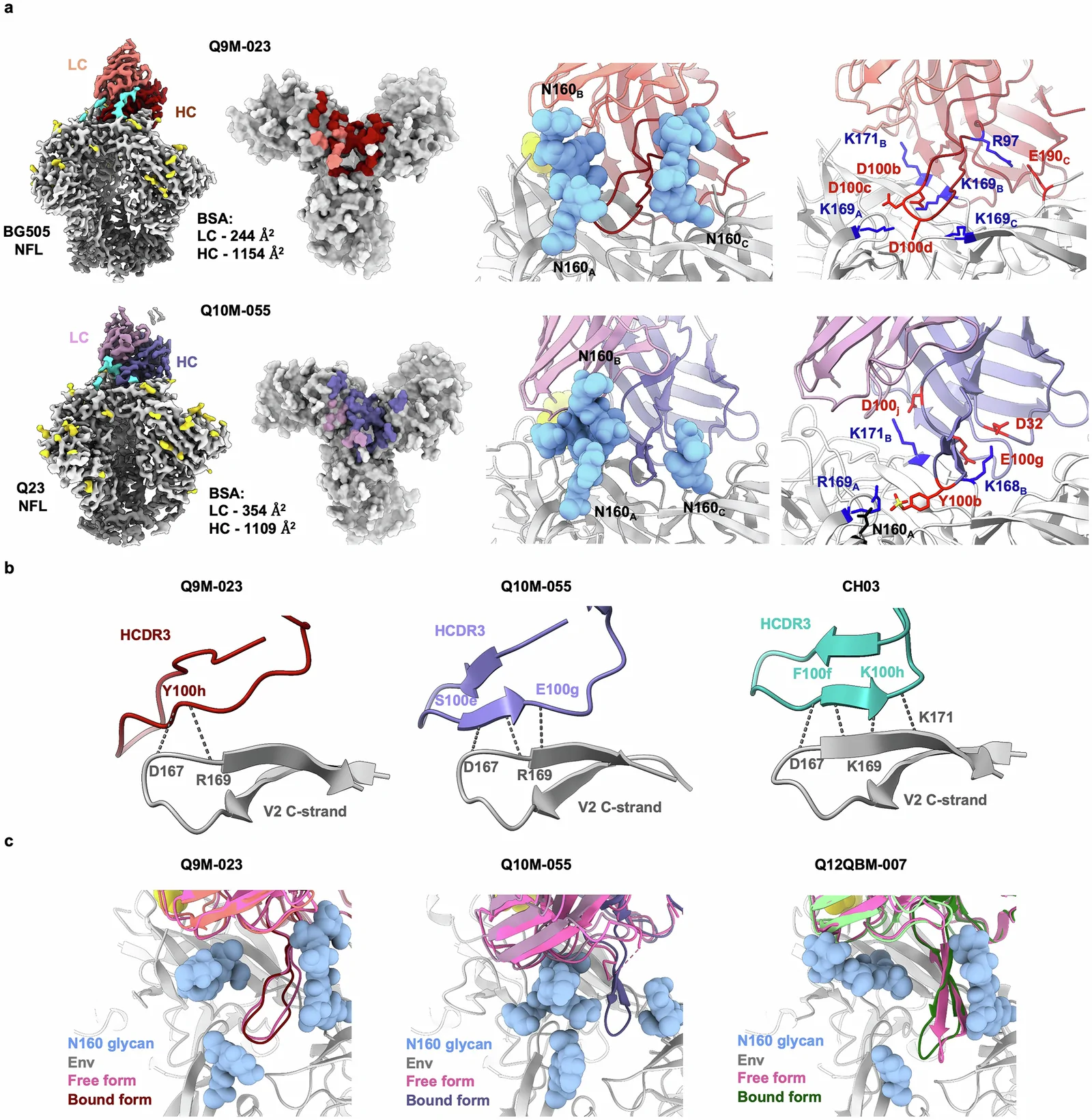
CryoEM complex structures of Q9M-023 and Q10M-055, interaction with V2 C-strand, comparison to the unliganded HCDR3. (**a**) CryoEM maps, epitope footprint, HCDR3 interactions with N160 glycan, and key contact residues of Q9M-023 in complex with BG505 NFL (top) and Q10M-055 in complex with Q23 NFL (bottom). N160 glycans are coloured blue, all other glycans are coloured yellow. Q10M-055 sulfated tyrosine (Y100b) is coloured in yellow and red (**b**) Interaction of HCDR3 with V2 C-strand, compared to bNAb CH03. Hydrogen bond interactions are shown as dotted lines. (**c**) Superposition of HCDR3 loops from (unliganded) crystal structures and those of the cryoEM complex structures.

We determined high-resolution crystal structures of four Fabs, Q9M-023, Q10M-055, Q12BBM-069 and Q12QBM-007, and observed well-ordered, protruding HCDR3 β-hairpin conformations in all of the mAbs except for that of Q10M-055 (Extended Data Fig. 5a and Supplementary Table 2). To define their epitope specificities at atomic-level detail, we analysed these cross-neutralizing mAbs in a complex with NFL trimers using high-resolution cryo-EM (Fig. 4a, Extended Data Fig. 6a,b and Supplementary Table 3). Cryo-EM maps and models of the two most broadly neutralizing mAbs, Q12BBM-069 and Q12QBM-007, in a complex with the BG505 NFL trimer, revealed that the vaccine-elicited mAbs bound a quaternary epitope at the trimer apex that greatly overlapped with that of the infection-induced human bNAb PG9 as shown in the BG505 SOSIP–PG9 complex structure (PDB: 7T77) (Fig. 4a). The epitopes of the NHP mAbs on the trimer surface revealed a similar overall Env contact footprint, especially for the heavy chains, relative to PG9, but with a larger buried surface area (Fig. 4a). The mAb–trimer interactions are dominated by β-hairpin HCDR3 loops that contact the glycans at position Asn160 in all three protomers—a pattern also seen with PG9 (Fig. 4a).

**Fig. 5:**
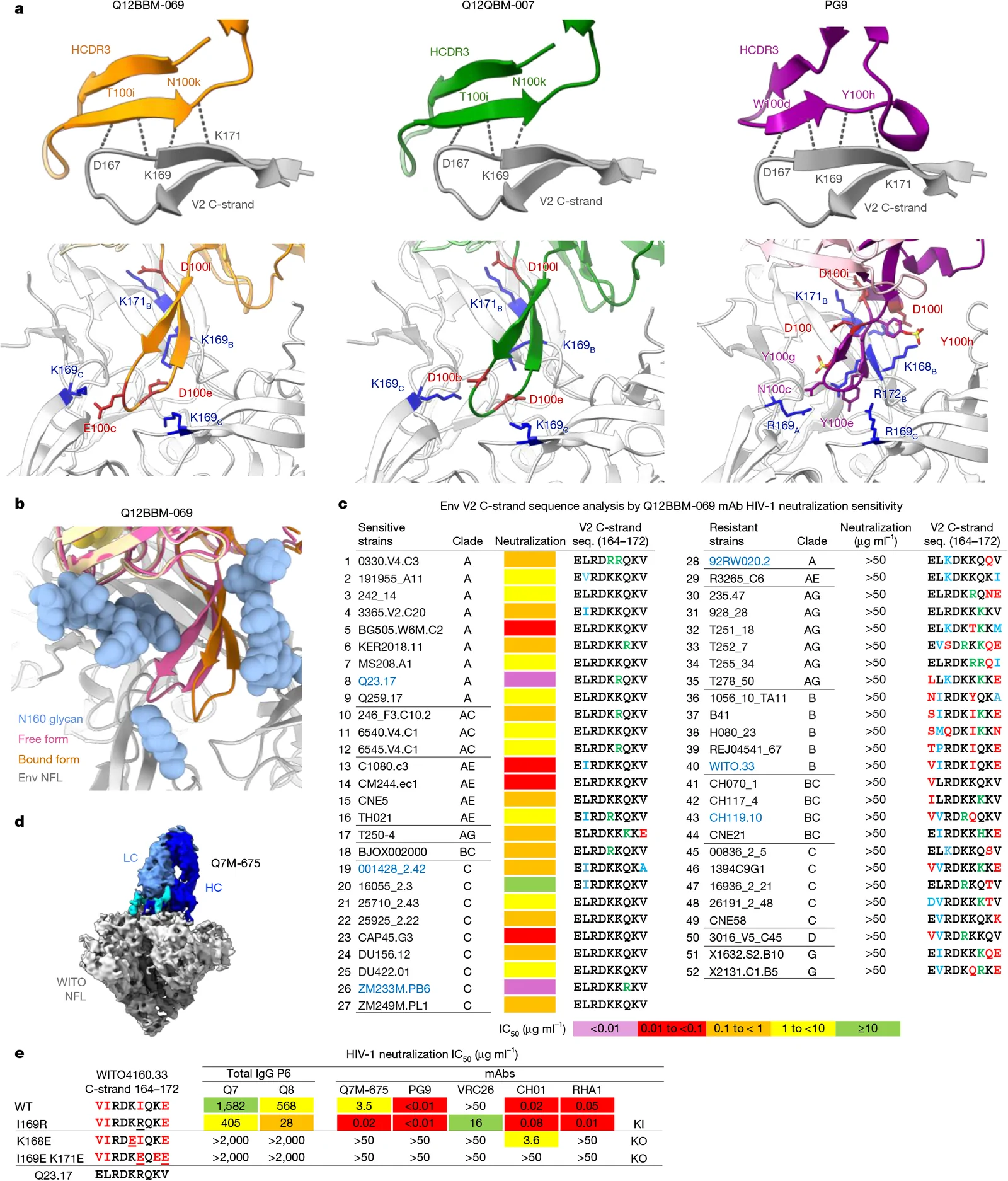
Interactions of the HCDR3 loops of mAbs with the V2 C-strand at the trimer apex. **a**, The HCDR3 β-hairpin loops of Q12BBM-069 and Q12QBM-007 interact with the V2 C-strand of Env through hydrogen bonding (shown as dotted lines). Similar interactions were observed for the bNAb PG9 (PDB: 7T77) (top). Bottom, key interactions of the HCDR3 tip with the Env trimer-apex. Antibody residue numbers followed by a lowercase letter denote insertions by the Kabat antibody reference numbering system. Env residues followed by a subscript uppercase letter define the protomeric gp120 subunit of the trimer. **b**, Superimposition of the unbound Q12BBM-069 Fab (X-ray) with the bound form (cryo-EM). **c**, V2 C-strand sequences of viruses neutralized by (1–27, left) and not neutralized (28–52, right) by Q12BBM-069. **d**, Cryo-EM density of the WITO NFL in a complex with macaque antibody Q7M-675. **e**, Neutralizing IC_50_ (µg ml^−1^) titres against HIV-1 WITO.33 viral variants including C-strand-knockout mutant viruses (bottom). Colour scale legend for serum IgG and mAbs is shown in Figs 2 and 3c, respectively. Neutralization assays were repeated twice with similar results.

To determine the importance of the antibody–Asn160 glycan interactions, we generated viruses lacking the Asn160 glycan by genetically modifying the corresponding potential N-linked glycosylation site. Neutralization of these viruses was heavily dependent on the presence of the Asn160 glycan, consistent with the apex-directed bNAbs^3,16,17^ (Fig. 4b). Similar binding patterns and Asn160 glycan interactions were observed in complexes of Q9M-023 and Q10M-055 with BG505 and Q23 NFL trimers, respectively (Extended Data Fig. 6a). The HCDR3s of all four mAbs adopted a β-hairpin motif that engaged the basic C-strand of Env V2, using both sidechain and backbone interactions, as described for the bNAbs PG9 and CH03 (Fig. 5a and Extended Data Fig. 6b). The acidic residues of the HCDR3 make electrostatic interactions with basic residues in the trimer apex (Fig. 5a, Extended Data Fig. 6a and Supplementary Table 4). In Q10M-055, a sulfated tyrosine, Tyr100b, at the tip of the HCDR3 interacts with the Arg169 sidechain and the asparagine of the Asn160 glycan, stabilizing its HCDR3 loop that is partially disordered in the unliganded crystal structure. This sulfated tyrosine–Env interaction occurs with the most distal Env protomer, increasing the antibody’s reach despite its shorter HCDR3 (Extended Data Fig. 6a (right)).

Cryo-EM structures of the Q12BBM-069 and Q12QBM-007 mAb–trimer complexes, compared with the crystal structures of the unliganded antibodies, reveal that the HCDR3 β-hairpins shift laterally to avoid steric clashes and facilitate interactions with the Asn160 glycan and nearby Env residues (Fig. 5b and Extended Data Fig. 6c). This shift varies by antibody, as measured by the Cα distance between equivalent residues at the tip of the loop—7.3 Å (Q12BBM-069), 2.8 Å (Q12QBM-007) and 2.3 Å (Q9M-023)—suggesting that the elicited antibodies may have been selected in part for their HCDR3 flexibility, especially Q12BBM-069.

## HIV Env V2 epitope mapping analysis

To better understand the neutralization specificities of the cross-reactive mAbs isolated here, we analysed the sequences of the HIV Env C-strand (residues 164–172) of the Asn130-glycan-lacking viruses tested in this study. The C-strand amino acid signature of viruses neutralized by Q12BBM-069, the most broadly neutralizing antibody isolated here, included the following residues Glu164, Leu/Ile/Val165, Arg166, Asp167, Lys/Arg168, Lys/Arg169, Gln/Arg/Lys170, Lys171 and Val/Ala/Glu172, or 164-ELRDKKQKV-172 as defined by the most frequent amino acid at each position (Fig. 5c). This V2 sequence is well represented in the NFL immunogens Q23, ZM233 and 001428 NFLs. The isolated mAbs make electrostatic interactions with basic amino acids (Lys/Arg) often present at positions 168, 169 and 171 (Fig. 5a and Extended Data Fig. 6a). This analysis reveals that divergence from the consensus 164-ELRDKKQKV-172 sequence, especially residues 168, 169 and 171, may provide natural resistance to Q12BBM-069 neutralization (Fig. 5c). There is natural resistance in multiple isolates reminiscent of that observed by the apex bNAb VRC26, which does not neutralize clade B viruses^13^. Envs with V2 C-strands that differed by two or more positions as defined by the identified signature, like that of the clade B WITO.33, are resistant to neutralization by Q12BBM-069. The WITO.33 C-strand amino acid sequence, 164-**VI**RDK**I**QK**E**-172, diverges substantially from that of the viruses neutralized by Q12BBM-069 (divergent residues are shown in bold). Notably, antibody Q7M-675 neutralized WITO.33, overcoming this sequence variability (Fig. 5d,e). Indeed, serum IgG from NHP Q7, from which Q7M-675 was isolated, and Q8, neutralized this clade B virus suggesting that the C-strand sequence divergence can be overcome (Fig. 5e). Mapping of the serum IgG from NHPs Q7 and Q8 demonstrated that the WITO.33 neutralizing activity is directed to the V2 C-strand. We generated C-strand-knockout viral variants that eliminate neutralization activity of apex bNAbs (a double mutant I169E/K171E and a single mutant K168E) (Fig. 5e). No neutralization activity was observed with either of these knockout viruses. By contrast, a single I169R substitution in the divergent WITO.33 V2 sequence increased the neutralization sensitivity to the Q7M-675 mAb and the Q7 and Q8 polyclonal IgG, highlighting the importance of residue 169 in the apex-targeted antibody response elicited in this study. Taken together, these results indicate that neutralization of the divergent clade B WITO.33 virus is directed primarily to the C-strand and that natural resistance afforded by sequence variability in this region can be overcome. This information will guide design of improved immunization regimens based on Env trimer-liposomes. Notably, the serum IgG from all trimer-liposome-immunized NHPs neutralized viruses containing divergent C-strand sequences (that is, CH115.12, Ce1086_B2, 00836.2.5, X2131.C1.B5), indicating the presence of alternative antibody lineages in these NHPs, either directed to the apex or, potentially, targeting other epitopes.

## Discussion

Here we describe a vaccine strategy using near-native NFL-stabilized Env trimers covalently arrayed on liposomes that elicited robust apex-directed cross-neutralizing antibody responses against HIV. Our priming immunogen, Q23 NFL, was identified based on its natural and rare ability to engage the germline-reverted apex-targeting bNAb RHA1. Sequential heterologous boosting of liposome-arrayed trimers generated cross-neutralizing antibody responses in all NHPs. After memory B cell sorting with native-like trimers, we isolated mAbs with long, negatively charged HCDR3s (20 residues or longer), typical of apex-targeting NHP bNAbs. We demonstrate that a subset of these mAbs cross-neutralizes a panel of clinical isolates representing different HIV clades. High-resolution cryo-EM structures of these antibodies in a complex with selected NFL trimers confirm that they target the apex of the HIV-1 Env trimer and display structural features with high similarity to the human-infection-elicited bNAbs PG9 and CH03.

These results substantially advance efforts in the HIV vaccine field to elicit broadly neutralizing antibody responses targeting the glycan-shielded HIV-1 Env trimer apex, beyond the priming of bNAb precursors alone. Typically, the bNAb apex precursors bind to the original engineered germline-targeted trimers but not to more native, wild-type trimers and fail to neutralize wild-type virus^40^. Using germline-identified, natural apex sequence trimers, the results presented here demonstrate that we not only prime apex-directed precursors, but we also educate B cells to take the next several steps by heterologous trimer boosting to drive affinity maturation resulting in apex-directed antibodies that cross-neutralize a wide array of tier-two multiclade clinical isolates. The antibodies elicited here recognize wild-type Env, bind to and neutralize the autologous viruses already after sequential prime:boosting with two NFL trimers (Q23 and ZM233), and then mature to develop cross-neutralizing activity after heterologous trimer boosting. We would assert that this approach is an alternative to—and, in this setting, may be preferable to—remodelling the immunogen to become high affinity by altering the Env-binding site to increase priming efficiency. Under such circumstances, achieving antibody wild-type Env recognition requires boosting energy to evolve and, in the best case, recognize the native Env trimer to achieve neutralization. By contrast, our vaccine strategy efficiently generates antibody responses capable of neutralizing autologous virus immediately after vaccination with the two tandem priming immunogens.

The molecular mimicry observed between the vaccine-elicited apex cross-neutralizing mAbs presented here and the infection-driven bNAbs validates our approach. The vaccine-elicited mAbs described in this study successfully engage the N-linked glycan at position 160, an important determinant in neutralization by bNAbs PG9, PGT145, CH01 and VRC26^24^. Extensive engagement of glycans in the HIV Env trimer by cross-neutralizing antibodies is rarely achieved by vaccination. The macaque antibody–Asn160 glycan interactions are probably necessary for neutralization and to extend the neutralization breadth and increase the potency. Neutralization primarily of Asn130-glycan-lacking viruses may be a result of the use of trimer immunogens that also lack this N-glycan, suggesting alternative strategies moving forward. The cryo-EM structures indicate that the Asn130 glycan might clash with the light chain of the macaque antibodies. However, this clash can be overcome as we demonstrate that some antibodies do neutralize some viruses possessing the Asn130 glycan. Thus, incorporation of the Asn130 glycan in future immunogens and probes may be beneficial to more efficiently elicit antibodies that can accommodate this glycan. We speculate that, using structure-guided immunogen modification, it is possible to expand the neutralization breadth by using a wider array of C-strand sequences to better evolve this process. In this regard, we detected neutralization of the clade B WITO.33 HIV strain by polyclonal serum IgG from two NHPs and we isolated an antibody (Q7M-675) with such activity, suggesting that this type of antibody evolution by vaccination is possible. The ability to elicit slightly different specificities in outbred animals shows the potential of the repertoire to elicit many such antibodies from the structural basics of the V2 apex signature with slightly different solutions and the potential to become bNAbs.

This proof-of-principle study establishes a template for further improvements using this vaccine-focused positive-control system that can be optimized for improvement by head-to-head single variable experiments in NHPs. Transitioning to humans can be accomplished similarly by using UCAs or germline-reverted bNAbs to select natural Env sequences that activate precursor B cells in the human antibody repertoire. With approximately 35 million people living with HIV-1 each with an extensive array of potentially transmissible quasi-species, a sequential heterologous Env trimer prime–boosting strategy such as the one described here is generally considered necessary to develop a broadly effective HIV vaccine. Owing to its extraordinary antigenic diversity, HIV cannot be addressed with a single, annually updated vaccine like those used for influenza or SARS-CoV-2. Rather, it will probably require a multi-dose immunization strategy aimed not at strain matching—which is infeasible—but at inducing broadly reactive antibodies able to counter the virus’s vast global diversity.

## Methods

### Animals

The animal work was approved by the Emory University Institutional Animal Care and Use Committee (IACUC) under protocol 202100136.

Twelve adult Indian-origin rhesus macaques (*Macaca mulatta*) (RM) were housed at the Emory National Primate Research Center (ENPRC) and maintained in accordance with NIH guidelines. Animal care facilities are accredited by the US Department of Agriculture (USDA) and the Association for Assessment and Accreditation of Laboratory Animal Care (AAALAC) International. Animals were treated with anaesthesia (ketamine, 5–10 mg per kg; or telazol, 3–6 mg per kg) and analgesics for procedures including intramuscular (i.m.) and subcutaneous (s.c.) immunization, and blood draws as per veterinarian recommendations and IACUC approved protocols. Rhesus macaques were male and female, aged 3–4 years at the start of the study, with an average weight of 4.8 kg. Animals were grouped to divide age and weight as evenly as possible between the groups receiving either soluble or liposome-conjugated trimers. Animals were housed in pairs for the duration of the study. For the immunization group size, we established the sample size primarily by extensive historical experience in immunization studies, which indicates that the assignment of six animals per immunization group will result in the detection of significant differences in antibody production among different experimental groups. Moreover, we have taken into consideration the three Rs (replacement, reduction and refinement) principle that guides the humane use of animals in scientific research; in particular, we considered reduction, which recommends using the fewest animals necessary to achieve valid experimental results.

### Cell lines

HEK293F cells were cultured in FreeStyle 293 Expression Medium (Thermo Fisher Scientific) supplemented with 1× antibiotic–antimycotic solution (Sigma-Aldrich) in a humidified incubator (125 rpm, 8% CO_2_, 37 °C). TZM-bl and HEK293T cells were cultured in Dulbecco’s modified Eagle medium (cDMEM) supplemented with 10% fetal BSA and 1× penicillin–streptomycin–glutamine (Gibco) at 5% CO_2_ and 37 °C. Expi293F cells were cultured in medium from 293F Expression System Kit in a humidified incubator (125 rpm, 8% CO_2_, 37 °C).

### Design of NFL trimer immunogens and probes

The Env sequences used for the generation of the NFL trimeric proteins were derived from the following HIV-1 viral sequences obtained from the Los Alamos National Laboratory HIV sequence database: Q23.17 (AF0048885.1), ZM233M.6 (DQ388517), WITO4160.33 (AY835451), 001428_2 (EF117266), 92RW020 (AY669706), BG505.W6M (DQ208456) and 16055_2 (EF117268). These DNA sequences were codon optimized to enhance protein expression and modified as follows to make stabilized NFL trimeric Env proteins: the natural HIV-1 leader sequence was replaced by a CD5 leader sequence. The four-residue furin cleavage site 508-REKR-511 was substituted with a ten-amino-acid flexible linker comprising the sequence G_4_SG_4_S^28^. The C terminus of the Env sequence was truncated at residue 664, resulting in the elimination of the membrane proximal external proximal region, transmembrane domain and cytoplasmic tail^41^. An additional linker comprising the residues GGGGSHHHHHHHHGSGC was added to the C terminus to facilitate coupling to liposomes through the terminal cysteine residue. Finally, a series of stabilizing mutations was introduced to create highly stable and homogeneous trimeric proteins with near-native conformation. These stabilizing mutations consist of the TD (BG505-trimer-derived residues), helix-breaking glycine and proline substitutions, unnatural disulfides and V3-loop and fusion-peptide-stabilizing mutations^29,30,32^.

### Trimer expression, purification, characterization and trimer-liposome preparation

The expression and purification protocols for HIV-1 Env NFL trimers were described in detail previously^28,30,42,43^. NFL trimers used as immunogens were expressed transiently in 293F cells and purified over a lectin column followed by negative selection using the non-neutralizing mAb F105 followed by size-exclusion chromatography (SEC). The trimers were characterized for their structural conformation and antigenicity by SEC, BLI and DSC. In brief, the BLI analysis was carried out on an Octet Red instrument (Sartorius) with IgGs immobilized on anti-human IgG Fc capture sensors (Sartorius). The Env trimers were assessed as free analytes in solution (PBS pH 7.4) at a final concentration of 250 nM. Association and dissociation were measured for 60 s respectively. The data were analysed using ForteBio software v.11.1 and kinetic parameters were obtained using a global 1:1 fit model. The thermal transition temperature (*T*_m_) of the NFL trimers was determined by DSC using a MicroCal VP-Capillary DSC instrument (Malvern Panalytics). The trimer samples were dialysed in PBS, pH 7.4, and 400 μl of the trimer sample at concentration of 0.25 mg ml^−1^ was loaded into the instrument. The dialysis buffer was used as the reference solution. The DSC experiments were performed at a scanning rate of 1 K min^−1^ under 3.0 atmospheres of pressure. The data were analysed after buffer correction, normalization and baseline subtraction using MicroCal VP-Capillary DSC analysis software provided by the manufacturer (Origin 7 SR4 v.7.0522). Liposome-conjugated trimers were used as immunogens in NHPs Q7–12. The conjugation protocol for generating trimer-liposomes was described in detail previously^31^. Liposomes comprised 1,2-distearoyl-*sn*-glycero-3-phosphocholine, cholesterol and 1,2-dipalmitoyl-*sn*-glycero-3-phosphoethanolamine-*N*-[4-(*p*-maleimidomethyl) cyclohexane-carboxamide]^31^. After chemical conjugation with the NFL trimers, the trimer-liposomes were further purified over the Superdex 200 Increase 10/300 GL column in PBS pH 7.4 buffer to remove unconjugated trimers from the trimer-liposomes. The conjugation of trimer to liposomes was confirmed by imaging the trimer-liposomes using negative-stain electron microscopy (Scripps Research, EM core).

### Calcium Flux

Spleen cells from the CH01 UCA dKI mice^44^ and the K46 B cell line expressing PG16^45^ were used for the calcium flux assay. CH01 UCA dKI cells were suspended at 4 million cells per ml in advanced DMEM, labelled with 1.5 μM Calbryte 520 AM (ATT Bioquest, 20651) and Pluronic F-127 (Invitrogen, P3000MP) for 30 min at 37 °C. CH01 UCA cells were washed with Advanced DMEM and stained with TruStain FcX PLUS (BioLegend, 156604) and Alexa Fluor 647 anti-mouse B220 (BioLegend, 103226) for 10 min at room temperature. After the staining protocol, the cells were washed with 2 mM CaCl_2_ HBSS. The cells were then incubated at room temperature for 30 min. Two million cells were aliquoted for flow cytometry analysis. Cells were stimulated with NFL trimers or trimer-liposomes and calcium signals were detected for 240 s measuring fluorescence at excitation/emission = 516 nm/533 nm (B2 peak channel) on the Cytek Aurora spectral flow cytometer (Cytek Biosciences). Analysis was performed using FlowJo (Becton Dickinson). For the PG16 K46 B cell line assay, surface expression of the BCR was induced by adding 1 μg ml^−1^ of doxycycline (Thermo Fisher Scientific, J6380506) 1 day before. The surface PG16 expression was confirmed by binding of FITC anti-human light chain lambda antibody (BioLegend, 316606) using the Cytek Aurora spectral flow cytometer (Cytek Biosciences). Further confirmation of PG16 surface expression was done using biotinylated Q23 NFL trimer conjugated to streptavidin-Alexa Fluor 647 (Molecular Probes, S32357). Trimer- or trimer-liposome-induced calcium flux was measured in a similar way as done for the CH01 UCA spleen cells.

### Immunization of NHPs

Twelve NHPs, divided into two groups of six, were inoculated at eight sites (four i.m. and s.c. bilateral immunizations on the deltoids and inner thighs) with 80 μg of Q23 NFL soluble trimer (NHPs Q1–Q6) or 80 μg of Q23 NFL trimer conjugated to trimer-liposomes (NHPs Q7–Q12) in 150 μg SMNP adjuvant. SMNP adjuvant was prepared as previously described^34^. NHPs were immunized with the same formulation (Q23 NFL) at weeks 2, 4, 6 and 8 as part of the divided-dose regimen. The NHPs were boosted with 150 μg of ZM233 NFL trimer in 375 μg SMNP adjuvant at week 20. Subsequent immunizations with heterologous trimers were performed at 31, 42, 64 and 102 weeks with 100 μg of trimer in 375 μg SMNP adjuvant. Pre-bleeds were collected before immunizations and test bleeds were collected on the day of immunizations and 14 days after each immunization. Blood was collected in Na-citrate CPT tubes (BD Biosciences) for PBMC and plasma isolation. Cells were cryopreserved in FBS with 10% DMSO (Gemini Bio, Fisher Bioreagents). Serum was collected with serum clot tubes (BD Biosciences).

### Neutralization

Serum IgG was affinity-purified with protein A Sepharose to approximately physiological levels (10 mg ml^−1^) as described previously^32^. Replication-incompetent HIV-1 Env pseudoviruses were produced by co-transfecting HEK293T cells with 15 µg of Env-deficient backbone plasmid (pSG3Δenv) and 5 µg HIV-1 Env plasmid in a ratio of 1:3 (total DNA:Fugene6 transfection reagent). Pseudoviruses were collected 72 h after transfection and incubation at 37 °C by centrifugation of cell culture supernatants at 3,000*g* for 10 min and stored at −80 °C. Inhibition of entry of HIV-1 Env pseudotyped viruses into standard TZM-bl cells was used to determine the neutralization capacity of sera, purified total serum IgG or mAbs as previously described in a half-well plate format^46^. TZM-bl cell suspension (50 µl; 110,000 cells per ml) in cDMEM was added to each well of white, flat-bottomed tissue culture treated plates 24 h before setting up the sample dilutions and incubated in a 37 °C CO_2_ incubator. In a 96-well U-bottomed plate, total serum IgG or antibodies were serially diluted in cDMEM five or six times (with 1:3 or 1:5 dilution factors) resulting in a total of six to seven dilution points. In a separate 96-well U-bottomed plate, the sample dilutions were mixed with prewarmed pseudovirus at a ratio of 1 to 5 (6 µl of sample dilution and 24 µl of pseudovirus), resulting in a maximal final total serum IgG concentration of 2 mg ml^−1^ or antibody concentration of 50 µg ml^−1^. The mixtures were then incubated at 37 °C for 1 h. cDMEM was then aspirated from the tissue culture plates containing TZM-bl cells, and 25 µl of the sample-pseudovirus mixture was pipetted directly onto the TZM-bl cells and placed into the 37 °C incubator for 24 h. After 24 h, 75 µl cDMEM was added to all wells of the plates and left at 37 °C for another 24 h. Finally, the cDMEM/sample mixes were aspirated from the plates, and the TZM-bl cells were lysed for 20 min on an orbital shaker at 400–500 rpm. Inhibition of entry was determined using the Promega Luciferase system, with luminescence detected using the Biotek NEO2M plate reader. The resulting Luciferase signals were measured in relative light units (RLU). The serum IgG or antibody concentrations that resulted in a 50% RLU reduction (IC_50_ values for purified IgG or mAbs) was determined by fitting the neutralization dose–response curves by nonlinear regression using a five-parameter Hill slope equation. Data were analysed using GraphPad Prism (v.10.2.1).

### Individualized immunoglobulin genotyping

To determine the germline V, D and J allele content of the studied rhesus macaques, full-length HC VDJ and LC VJ amplicons were generated as described previously^36^ and analysed using IgDiscover (https://gkhlab.gitlab.io/igdiscover22/). In brief, total RNA was extracted from approximately 5–10 million PBMCs using the Qiagen RNeasy mini kit. Then, 300 ng RNA was subjected to reverse transcription with gene-specific primers for the IgM, IgK and IgL constant regions using the Sensiscript RT kit (Qiagen), the cDNA was purified using the Qiagen MinElute PCR Purification Kit and was eluted in 20 µl of elution buffer. Then, 3 µl cDNA was subjected to the library PCR with 25× cycles using multiplex forward VH, VK and VL primer sets, and gene-specific primers for the IgM, IgK and IgL constant regions as previously described^36^. The libraries were gel-purified using the Qiagen MinElute Gel Extraction Kit and indexed with 10× PCR cycles according to the instructions of the Illumina MiSeq 2 × 300 bp kit. Indexed library PCR products were purified using the MinElute PCR Purification Kit, followed by AMPure XP magnetic bead (Beckman Coulter) purification before sequencing using the Illumina MiSeq 2 × 300 bp kit. The libraries were analysed using IgDiscover^37^ and Corecount^35^ to generate HC V, D and J, and LC V and J genotypes. Input databases for the IgDiscover analysis were KIMBD (http://kimdb.gkhlab.se/) for the HC alleles and IMGT for the LC alleles.

### Env-specific memory B cell sorting by flow cytometry

To produce Env trimer probes for B cell sorting, 10 µg of biotinylated Q23, BG505 and 16055 NFL trimers were conjugated to streptavidin–APC (Invitrogen) or streptavidin–BV421 (BioLegend) in five sequential steps, each incubation proceeded for 20 min at 4 °C. Frozen single-cell suspensions from blood mononuclear cells (PBMCs) from animals Q7, Q9, Q10 and Q12 were thawed at 37 °C, washed twice in prewarmed RPMI 1640 medium (HyClone) supplemented with 10% FBS (HyClone) and penicillin–streptomycin (100 IU ml^−1^, 100 µg ml^−1^) (Gibco). The cells were washed with PBS (Sigma-Aldrich) and counted using Trypan Blue exclusion of dead cells by a Countess II cell counter (Thermo Fisher Scientific). Cells were suspended in PBS and incubated for 30 min at 4 °C with Live/Dead Fixable Aqua Dead Cell Stain Kit (Life Technologies) according to the manufacturer’s instructions. Cells were washed with FACS buffer (PBS + 1% FBS) and surface stained with the following antibodies: CD3 FITC (SP34-2), CD14 FITC (M5E2), CD20 BV421 or PerCP-Cy5.5 (2H7), CD27 PE-Cy7 (M-T271), IgG PE-CF594 (G18-145) (all from BD Biosciences). Staining was performed for 30 min at 4 °C. After washing with FACS buffer, cells were subsequently stained with the fluorescently conjugated NFL trimers. Live CD3-CD14^−^CD20^+^CD27^+^IgG^+^ENV^+^ single cells were sorted on a four-laser FACSAria Fusion cell sorter (Becton Dickinson) into 96-well PCR plates (Eppendorf) containing 4 µl per well of ice-cold cell lysis buffer (0.5× PBS, 10 mM DTT and 2 U µl^−1^ RNAsin (all from Thermo Fisher Scientific)). After sorting, the 96-well plates were centrifuged, sealed and immediately frozen on dry ice and stored at −80 °C until use.

### Single B cell RT–PCR and mAb cloning

For cDNA synthesis the 96-well plates, containing single B cells, were thawed on ice. The reverse transcription (RT) was performed using SuperScript IV reverse transcriptase (Thermo Fisher Scientific) using random hexamers, oligodT, dNTPs (Invitrogen), Igepal CA-630 (Sigma-Aldrich) and RNAsin (Thermo Fisher Scientific). IgG HC and LC V(D)J sequences were amplified separately in 20 μl nested PCR reactions using 4 μl of cDNA for HC and 3 μl for LC in the first-round PCR and 1 μl PCR product in the second-round PCR using KAPA HiFi HotStart ReadyMix 2× (Roche). PCR products from positive wells were purified, Sanger sequenced (Genewiz) and analysed. HC and LC V(D)J sequences were cloned into expression vectors containing the human IgG1, Igκ1 or Igλ2 constant regions^47^. The sequences, engineered with overhangs complementary to the linearized vector ends, were assembled using the Gibson Assembly Master Mix (New England Biolabs). The reaction mixture, consisting of 50 ng of vector and 30 ng of insert in 20 μl reaction mix, was incubated at 50 °C for 1 h. After incubation, 1 µl of the diluted (1:3) reaction mix was transformed into XL10-Gold ultracompetent cells (Agilent Technologies) by heat shock at 42 °C for 30 s. Screening of transformed colonies was assessed by PCR, positive clones were expanded and plasmids were isolated using the Plasmid Plus Midi Kit (Qiagen). The correct sequences were confirmed using Sanger sequencing (Genewiz).

### mAb expression and purification

mAbs were expressed by co-transfecting equal amounts of HC and LC plasmids (18 µg each) into 30 ml FreeStyle 293-F cells (Thermo Fisher Scientific) (HEK293-F) cultured in FreeStyle 293 Expression Medium (Thermo Fisher Scientific) supplemented with 1× antibiotic–antimycotic solution (Sigma–Aldrich) at a density of 1.2 million cells per ml at >95% viability. Cultures were maintained in a humidified shaking incubator (125 rpm, 8% CO_2_, 37 °C). Transfections were carried out with FreeStyle Max reagent (Invitrogen) in Opti-MEM medium (Gibco). Then, 7 days after transfection, the mAbs were purified from the supernatant using Protein G Sepharose columns (Cytiva). Next, 4 μg of each purified antibody was analysed using SDS–PAGE under reducing conditions using NuPAGE 4–12% Bis-Tris polyacrylamide gels (Invitrogen).

### ELISA

Ninety-six-well plates (Half area Corning) were coated with lectin (Galanthus Nivalis Lectin, Vector laboratories) at 2 μg ml^−1^ for 1 h. The plates were blocked for 1 h with 150 μl PBS buffer comprising 2% non-fat milk and 5% FBS. The plates were washed four times with 150 μl of PBS buffer supplemented with 0.02% Tween-20 between each of the subsequent incubation steps. Soluble trimer or trimer-liposomes at 2 μg ml^−1^ were captured on the plates for 1 h. The plates were incubated for 1 h with fivefold serial dilutions of selected mAbs with a starting concentration of 50 μg ml^−1^. Antibody binding was detected using horseradish-peroxidase-conjugated anti-human IgG secondary antibody (Jackson ImmunoResearch) at a dilution of 1:5,000 for 1 h, followed by another wash and developed with TMB (Life Technologies) substrate solution. The plates were visually examined for development of colour, and the reaction was stopped with 0.3 N sulfuric acid, and the absorbance was measured at 450 nm. Data were plotted using GraphPad Prism v.9.4.1.

### EMPEM analysis

Total serum IgG was digested to polyclonal Fab using papain according to the manufacturer’s instructions (Pierce Fab Preparation kit, Thermo Scientific). For each immune complex, 1 mg of polyclonal Fab was incubated with 15 µg of Env NFL trimer overnight and purified the next morning using the Cytiva Superdex 200 Increase size-exclusion column. Fractions corresponding to immune complexes were pooled and adsorbed onto 400-mesh carbon-coated copper grids (Electron Microscopy Sciences) at a concentration of around 0.02 mg ml^−1^ for 10 s before blotting off the excess liquid. Grids were stained for 45 s using 2% (w/v) uranyl formate (Electron Microscopy Sciences) before blotting. Imaging was performed using a Thermo Fisher Scientific Glacios transmission electron microscope operating at 200 kV, equipped with the Thermo Fisher Scientific Falcon 4 camera (×73,000 magnification, 1.89 Å pixel size). Automated data collection was performed using EPU (Thermo Fisher Scientific) and data processing was performed using Relion (v.4.0)^48^, according to standard procedures for reference-free particle picking and 2D classification. After three rounds of 2D classification, particles from classes corresponding to immune complexes were subjected to 3D refinement with *C*_3_ symmetry and a 40 Å low-pass-filtered map of HIV Env ectodomain as the initial model. The initial model is based on PDB coordinates 6V0R, converted to a map using the molmap feature in UCSF ChimeraX^49^. After 3D refinement, *C*_3_ symmetry expansion was applied to the particles and 7 separate focused 3D classification skip align jobs were run (*K* = 10), each with a 40 Å diameter spherical mask over key HIV Env epitopes. The names of the epitopes and reference structures used for orienting the masks are: (1) gp41-base (PDB: 7L8Z), (2) gp41-GH (PDB: 7L8U), (3) gp41-FP (PDB: 7L8T), (4) gp120-GH (PDB: 7L8B), (5) C3V5 (PDB: 7L8Y), (6) CD4bs and gp120 interface (PDB: 7L8X), (7) V1V3 (PDB: 7L8E) and (8) V2-apex (PDB: 9D1W). For each epitope 3D classification, classes with visible Fab density were selected and subjected to 3D refinement, 2D classification and a second round of 3D classification. If the 3D refinement resulted in partial Fab density relative to the Env trimer, classes were selected from the subsequent round of 3D classification, and this was repeated until the reconstruction improved, or no change was noted. Final reconstructions were visually inspected and assigned to the correct epitope label. The number of final particles belonging to each epitope was divided by the total number of particles in the initial (*C*_3_) 3D refinement. This value, which can range from 0 to 3 due to *C*_3_ symmetry expanded particles used in the 3D classification steps, is described as the EMPEM magnitude. It is also assumed that most epitopes are represented three times on an Env trimer. An exception is made for the V2-apex epitope, and the EMPEM magnitude is normalized using a 3× multiplier, as V2-apex bNAbs are known to bind only at a ratio of 1 Fab per trimer. The total magnitude is the sum of each individual epitope EMPEM magnitude for a given animal and timepoint. Representative maps have been deposited to the Electron Microscopy Data Bank.

### Purification and crystallization of unliganded Fabs

Fabs were expressed in Expi293F cells (Thermo Fisher Scientific, A14527) transiently transfected with plasmid DNA encoding the Fab heavy and light chains at a 1:1 ratio. Transfections were performed using the ExpiFectamine 293 Transfection Kit in Opti-MEM medium. Then, 6 days after transfection, the culture supernatants were collected and sterile-filtered through a 0.22-µm membrane filter. Fabs were purified using the CaptureSelect CH1-XL affinity matrix (Thermo Fisher Scientific, 1943462050), followed by SEC on the Superdex 200 16/600 column equilibrated with 20 mM Tris, 150 mM NaCl, pH 7.5. The fractions corresponding to Fab proteins were pooled and concentrated to 12 mg ml^−1^ for crystallization. Crystallization screening was performed using our Rigaku CrystalMation robotic system with JCSG Core Suites 1–4, and Top96 Cryo screens at 20 °C. Crystals were cryoprotected, if necessary, by supplementing the reservoir solution with 15% ethylene glycol and then flash-cooled in liquid nitrogen for storage prior to data collection.

### X-ray data collection and structure determination

X-ray diffraction data were collected at beamline 17-ID-1 (AMX) of the National Synchrotron Light Source II at 100 K using a wavelength of 0.9197 Å. Crystals of Fabs Q9M-023, Q10M-055, Q12QBM-007 and Q12BBM-069 diffracted to resolutions of 2.34 Å, 1.63 Å, 1.54 Å and 1.79 Å, respectively. The crystallization conditions were as follows: Q9M-023 Fab crystallized in 40% MPD, 0.1 M cacodylate buffer at pH 6.5 and 5% (w/v) PEG-8000; Q10M-055 Fab in 100 mM HEPES at pH 7.5, 10% (v/v) ethylene glycol and 20% (w/v) PEG-8000; Q12QBM-007 Fab in 50% PEG-200 and 0.1 M citrate at pH 5.5; and Q12BBM-069 Fab in 1.26 M ammonium sulfate and 0.1 M cacodylate at pH 6.5. Data processing, including indexing, integration and scaling, was performed using autoPROC^50^. Structure determination was carried out by molecular replacement using Phaser within the Phenix software suite^51^, with an initial model generated by AlphaFold 3^52^. Subsequent model building and refinement were performed through iterative cycles using Coot and Phenix.refine, respectively^53,54^. The quality of the final structures was assessed using MolProbity^55^, and additional validation was carried out through the PDB validation server. Ramachandran statistics showed that 97.7%, 97.4%, 98.0% and 97.5% of residues for Q9M-023, Q10M-055, Q12BQM-007 and Q12BBM-069, respectively, were in favoured regions, with the remaining 2.3%, 2.6%, 2.0% and 2.5% in allowed regions. Data collection and refinement statistics are summarized in Supplementary Table 2.

### Cryo-electron microscopy

All Fabs used for structural analysis were prepared by cleaving the IgG using papain (Thermo Fisher Scientific, 44985). The following four immune complexes were incubated overnight: (1) 0.2 mg of BG505 NFL TD CC3+ with 0.25 mg of either Q12BBM-069, Q12QBM-007 or Q9M-023; (2) 0.2 mg of Q23 NFL TD CC3+ with 0.25 mg Q10M-055. Complexes were purified the next morning using the HiLoad 16/600 Superdex 200 pg (Cytiva) gel-filtration column. The fractions corresponding to each immune complex were pooled and concentrated using an Amicon 100 kDa MWCO centrifugal device to between 4 and 6 mg ml^−1^. A complex of Q7M-675 Fab with WITO NFL CC3+ was prepared without the gel-filtration column step to account for the lower affinity expected based on the weaker neutralization titre. The samples were vitrified using the Vitrobot Mark IV (Thermo Fisher Scientific). The temperature was set to 4 °C and the humidity was maintained at 100% during the freezing process. The blotting force was set to 1 and the wait time was set to 10 s. The blotting time was varied from 3 to 6 s. Detergent lauryl maltose neopentyl glycol (Anatrace) or octyl-β-glucoside (Anatrace) was added to the sample to a final concentration of 0.005 mM or 0.1% (w/v), respectively, shortly before freezing. UltrAuFoil 1.2/1.3 (Au, 300-mesh; Quantifoil Micro Tools) grids were used and glow discharged (PELCO easiGlow, Ted Pella) for 40 s before sample application. Then, 0.5 µl of detergent was mixed with 3.5 µl of the samples and 3 µl of the mixture was immediately loaded onto the grid. After blotting, the grids were plunge-frozen into liquid-nitrogen-cooled liquid ethane.

The samples were loaded into a Thermo Fisher Scientific Glacios 2 TEM operating at 200 kV equipped with a Thermo Fisher Scientific Falcon 4i direct electron detector. The exposure magnification was set to ×190,000 with a pixel size at the specimen plane of 0.718 Å. EPU software (Thermo Fisher Scientific) was used for automated data collection. Micrograph video frames were motion corrected, dose weighted and CTF correction was performed using cryoSPARC Live^56^. cryoSPARC was used for the remainder of data processing. Particle picking was performed using blob picker initially followed by template picker. Particles were initially down-sampled by a factor of 4 and multiple rounds of 2D classification were performed followed by 3D ab initio reconstruction. The remaining particles were then re-extracted, with optional Fourier cropping to reduce the memory requirements of large box sizes, resulting in image pixel sizes of 1.034 Å (Q12BBM-069 + BG505; Q10M-055 + Q23), 1.005 Å (Q12QBM-007 + BG505) or 0.718 Å (Q9M-023 + BG505; Q7M-675 + WITO). 3D non-uniform refinement was performed. For all cryo-EM maps, the global resolution was estimated using half maps and a Fourier shell correlation cut-off of 0.143, and the local resolution was estimated using the cryoSPARC Local Resolution tool with 0.143 FSC cut-off. Relatively lower resolution (4.3 Å global FSC estimate), due to preferred orientation and low amounts of Fab-bound trimers, prevented accurate model building into the Q7M-675 + WITO complex. Initial models were generated using AlphaFold3 and docked into the cryo-EM maps using UCSF ChimeraX^49,52^. Manual building was performed in Coot v.0.9.8 and real space refinement in Phenix^57,58^. Final models were validated using MolProbity and EMRinger in the Phenix suite. Buried surface area calculations and interface analyses were performed using UCSF ChimeraX. Data collection, processing and model statistics are summarized in Extended Data Fig. 5 and Supplementary Table 3.

### Reporting summary

Further information on research design is available in the Nature Portfolio Reporting Summary linked to this article.

## Online content

Any methods, additional references, Nature Portfolio reporting summaries, source data, extended data, supplementary information, acknowledgements, peer review information; details of author contributions and competing interests; and statements of data and code availability are available at 10.1038/s41586-026-10429-3.

## Supplementary information

Supplementary Information Supplementary Fig. 1 and Supplementary Tables 1–4. Supplementary Fig. 1: FACS gating strategy. Supplementary Table 1: sort statistics and antibody genetics. Supplementary Table 2: X-ray data collection and refinement statistics. Supplementary Table 3: cryo-EM data collection and refinement statistics. Supplementary Table 4: antibody heavy and light chains, Env contacts and somatic hypermutation.

Reporting Summary

Peer Review File

## Source data

Source Data Fig. 1

Source Data Fig. 3

Source Data Extended Data Fig. 1

## Data Availability

The HC VDJ and LC VJ sequences of the Env-specific mAbs have been deposited in GenBank as follows: for the heavy chains: PX281429–PX281473 and PX717267–PX717279; for the kappa chains: PX281474–PX281512 and PX717254–PX717264; and for the lambda chains: PX281513–PX281518 and PX717265–PX717266. IgM, IgK and IgL repertoire data are available from ENA under the accession numbers ERR16022197 (Q7 IGM), ERR16022198 (Q7 IGK), ERR16022199 (Q7 IGL), ERR15498913 (Q9 IGM), ERR15498914 (Q9 IGK), ERR15498915 (Q9 IGL), ERR15498916 (Q10 IGM), ERR15498917 (Q10 IGK), ERR15498918 (Q10 IGL), ERR15498919 (Q12 IGM), ERR15498920 (Q12 IGK) and ERR15498921 (Q12 IGL). Cryo-EM maps have been deposited in the Electron Microscopy Data Bank (EMDB) under accession codes EMD-72009, EMD-72031, EMD-72033, EMD-72035 and EMD-74449, and cryo-EM models have been deposited in the Protein Data Bank (PDB) under accession codes 9PY5, 9PYD, 9PYK and 9PYH. X-ray crystal structures of the Fabs have been deposited in the PDB under the codes 9PYN, 9PYY, 9PZ2 and 9PZ3. Representative negative-stain EMPEM maps have been deposited into the Electron Microscopy Data Bank under accession codes EMD-72735, EMD-72736, EMD-72737, EMD-72738 and EMD-72739. All deposited data are publicly available. Any additional information required to reanalyse the data reported in this Article is available from the corresponding authors on request. Source data are provided with this paper.

## Code availability

No custom code was used in the generation of the data in this Article. The IgDiscover software is available online (https://gkhlab.gitlab.io/igdiscover22/).
