# Supplementary material for: Vaccination generates broadly cross-neutralizing antibodies to the HIV Env apex
Source: Nature. 2026 Apr 29;654(8119):777–85. doi: 10.1038/s41586-026-10429-3 (PMC13275315; doi:10.1038/s41586-026-10429-3)
Supplement: Supplementary file 1 — Supplementary Fig. 1 and Supplementary Tables 1–4. Supplementary Fig. 1: FACS gating strategy. Supplementary Table 1: sort statistics and antibody genetics. Supplementary Table 2: X-ray data collection and refinement statistics. Supplementary Table 3: cryo-EM data collection and refinement statistics. Supplementary Table 4: antibody heavy and light chains, Env contacts and somatic hypermutation. [file 41586_2026_10429_MOESM1_ESM.pdf]

nature portfolio

https://doi.org/10.1038/s41586-026-10429-3

Supplementary information

Vaccination generates broadly cross-neutralizing antibodies to the HIV Env apex

In the format provided by the authors and unedited

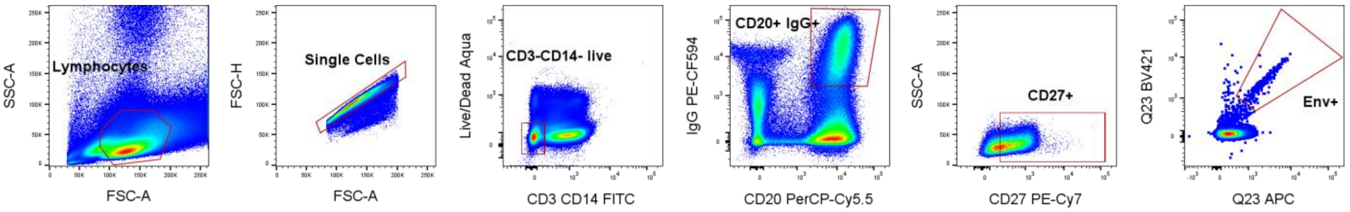

Supplementary Figure 1. FACS gating strategy

FACS gating strategy to isolate Env-specific macaque memory B cells from PBMCs. In brief, macaque PBMCs were first gated on lymphocytes based on FSC-A/SSC-A, followed by FSC-A/FSC-H to exclude doublets/select singlets. Dead cells, T cells, and monocytes were excluded using Aqua Live/Dead staining and CD3 CD14 markers, respectively. IgG^+ B cells were defined as $CD20^+IgG^+$. Env-specific memory B cells were identified as $CD27^+Env^+$ (Q23, BG505, 16055) within the IgG^+ B cell population.

Supplementary Table 1a. Sort statistics

	sampling time point	probe	stained PBMCs (million)	sorted cell number	productive HC+LC pairs	mAb ID
Q7	P6	Q23	8.75	376	208	Q7M-450[$$$], Q7M-526, Q7M-562, Q7M-653, Q7M-675, Q7M-773[$$$]

	sampling time point	probe	stained PBMCs (million)	sorted cell number	productive HC+LC pairs	mAb ID
Q9	P4	Q23	5	44	16	Q9M-001, Q9M-009[****]
	P5	Q23	9.1	125	64	Q9M-023[****], Q9M-213[****], Q9M-218[****]
	P6	Q23	7.4	188	137	Q9M-092, Q9M-225[****], Q9M-229[****], Q9M-231[****], Q9M-246[****], Q9M-247[****]

	sampling time point	probe	stained PBMCs (million)	sorted cell number	productive HC+LC pairs	mAb ID
Q10	P4	Q23	5.85	234	112	Q10M-054[*], Q10M-055[*], Q10M-061[*], Q10M-092[*], Q10M-113[*], Q10M-100[**], Q10M-123[**], Q10M-048[#], Q10M-103[#], Q10M-083, Q10M-112, Q10M-122, Q10M-137
	P5	Q23	2.13	94	47	Q10M-209

	sampling time point	probe	stained PBMCs (million)	sorted cell number	productive HC+LC pairs	mAb ID
Q12	P4	Q23	2.7	36	10	Q12M-031[***], Q12M-032[***], Q12M-035
	P5	16055	1.8	41	33	Q1216M-031[§§]
	P5	16055+BG505	0.5	12	9	Q12B16M-007[§§]
	P5	BG505	11.4	119	80	Q12BBM-069[##], Q12BBM-023[***], Q12BBM-068[***], Q12BBM-045[¤], Q12BBM-056[¤], Q12BBM-065[¤], Q12BBM-085[¤], Q12BBM-042[###], Q12BBM-076[###], Q12BBM-022[$], Q12BBM-026[$], Q12BBM-044[$], Q12BBM-043
	P5	Q23+BG505	9	94	40	Q12QBM-007[***], Q12QBM-034[¤]
	P6	Q23	8.8	376	221	Q12M-061[##], Q12M-165[##], Q12M-131[§], Q12M-158[§], Q12M-197[§], Q12M-190[&], Q12M-283[&]

Supplementary Table 1b. Antibody genetics

NHP ID	mAb ID	lineage name	IGHV allele	IGHD allele	IGHJ allele	HCDR3	% VH SHM nt	% VH SHM aa	HCDR3 length	IGKV/IGLV allele	IGKJ/IGLJ allele
Q7	Q7M-675		IGHV4-NL_22*01_S6977	IGHD3-15*01	IGHJ5-4*01	VRDTENYYPPDDYDYYYTGYNRFDV	13.2	25	25	IGKV1-NL_16*01_S1046_kim	IGKJ4*01
	Q7M-562		IGHV4-NL_17*01_S2469	IGHD3-15*01	IGHJ5-4*01	ARGASYYDDDDGYYYSEDRFDV	7.3	12.0	22	IGKV1-25*02_kim	IGKJ4*01
	Q7M-773	Q7M-450	IGHV2-7*02_S1732	IGHD3-15*01	IGHJ3-2*01	ARSGGLFYEDDYGYYPAMEDDAFDF	5.7	11.1	25	IGLV2-38*01_S7244_kim	IGLJ1*01
	Q7M-450		IGHV2-7*02_S1732	IGHD3-15*01	IGHJ3-2*01	ARSGGLFYEDDYGYYPAKEDDAFDF	4.7	10.1	25	IGLV2-38*01_S7244_kim	IGLJ1*01
	Q7M-653		IGHV4-NL_17*01_S0147	IGHD3-15*01	IGHJ1-1*01	ARGARYYEDDSGYYYSRYFDL	5.3	11.0	21	IGKV1-94*01_S1549_kim	IGKJ4*01
	Q7M-526		IGHV4-150*01_S3825	IGHD3-15*01	IGHJ4-3*01	ARGASFYEDARGASFYEDEYGYFYTSPLPDF	3.4	4.1	31	IGKV1-94*01_S0326_kim	IGKJ4*01
Q9	Q9M-246	Q9M-023	IGHV4-117*01	IGHD3-15*01	IGHJ3-2*01	ASPIRVYYEEDDDYYYTYEDAFHV	9.2	13.4	24	IGKV1-21*01_kim	IGKJ2*01
	Q9M-247		IGHV4-117*01	IGHD3-15*01	IGHJ3-2*01	ARPVRADHEDDDGYYYSYDDAFDL	11.2	18.4	24	IGKV1-21*01_kim	IGKJ2*01
	Q9M-225		IGHV4-117*01	IGHD3-15*01	IGHJ3-2*01	ARPMRVDYEEDDEYYYMYEDAFDI	7.5	11.2	24	IGKV1-21*01_kim	IGKJ2*01
	Q9M-229		IGHV4-117*01	IGHD3-15*01	IGHJ3-2*01	ARPVRADHEDDDGYYYSYDDAFDL	11.6	18.4	24	IGKV1-21*01_kim	IGKJ2*01
	Q9M-023		IGHV4-117*01	IGHD3-15*01	IGHJ3-2*01	ARPVRADHEDDDGYYYSYDDAFDL	8.8	13.3	24	IGKV1-21*01_kim	IGKJ2*01
	Q9M-009		IGHV4-117*01	IGHD3-15*01	IGHJ3-2*01	ARPIRVHYEDDDEYYYMYEDAFDL	8.2	16.3	24	IGKV1-21*01_kim	IGKJ2*01
	Q9M-231		IGHV4-117*01	IGHD3-15*01	IGHJ3-2*01	GRPIRVDYDDDDEYYYMYEDAFEI	9.5	15.3	24	IGKV1-21*01_kim	IGKJ2*01
	Q9M-218		IGHV4-117*01	IGHD3-15*01	IGHJ3-2*01	ARPMRVDYEEDDEYYYMYEDAFDL	7.1	10.2	24	IGKV1-21*01_kim	IGKJ2*01
	Q9M-213		IGHV4-117*01	IGHD3-15*01	IGHJ3-2*01	ARPIRVDYEDDDDYYYMYEDAFEF	10.5	17.3	24	IGKV1-21*01_kim	IGKJ2*01
	Q9M-092		IGHV4-117*01	IGHD3-15*01	IGHJ6-6*01	AREGPRVDEDDGFYHYTSALDA	7.4	14.3	22	IGKV3-NL_6*01_S4875_kim	IGKJ2*01
	Q9M-001		IGHV4-144*01_S3113	IGHD3-15*01	IGHJ5-4*03	ATTSPYYEEDSGYYYTDDRFDV	10.2	18.4	22	IGKV1-NL_10*01_S1234_kim	IGKJ3*01
Q10	Q10M-055	Q10M-055	IGHV4-117*01_S8404	IGHD3-15*01	IGHJ3-2*01	ASDLYYEDDYGYSYEGLDVFDF	8.8	16.3	22	IGKV3-42*02_kim	IGKJ1*01
	Q10M-054		IGHV4-117*01_S8404	IGHD3-15*01	IGHJ3-2*01	ASDLYYEDDDGYSYEGLDVFDF	12.8	19.4	22	IGKV3-42*02_kim	IGKJ1*01
	Q10M-061		IGHV4-117*01_S8404	IGHD3-15*01	IGHJ3-2*01	ARDIYYEDDDGYSYTGLDVFDF	7.4	13.3	22	IGKV3-42*02_kim	IGKJ1*01
	Q10M-092		IGHV4-117*01_S8404	IGHD3-15*01	IGHJ3-2*01	ASDLYYEDDYGVSYAGLDVFDF	8.4	14.3	22	IGKV3-42*02_kim	IGKJ1*01
	Q10M-113		IGHV4-117*01_S8404	IGHD3-15*01	IGHJ3-2*01	ASDLYYEDDYGYSYAGLDVFDF	7.1	14.3	22	IGKV3-42*02_kim	IGKJ1*01
	Q10M-100	Q10M-100	IGHV4-117*01	IGHD3-15*01	IGHJ5-4*02	ARDGTYYDDDDGYYYTDQWFNV	8.8	16.3	22	IGKV3-42*02_kim	IGKJ1*01
	Q10M-123		IGHV4-117*01	IGHD3-15*01	IGHJ5-4*02	ARDGTYYDDDDGYYYTDNWFNV	10.1	15.3	22	IGKV3-42*02_kim	IGKJ1*01
	Q10M-048	Q10M-048	IGHV4-93*01	IGHD3-15*01	IGHJ4-3*01	VRDVSGRDYEGDDGYYFGRFFDF	7	12.1	23	IGKV1-NL_16*01_S1046_kim	IGKJ1*01
	Q10M-103		IGHV4-93*01	IGHD3-15*01	IGHJ4-3*01	VREISGPDYEDDDGYYFGRYFDH	5	8.1	23	IGKV1-NL_16*01_S1046_kim	IGKJ1*01
	Q10M-083		IGHV4-117*01	IGHD3-15*01	IGHJ5-4*02	ARDGEFYDDDDDSSFTQDWFDV	8.4	16.3	22	IGKV3-12*02_kim	IGKJ4*01_S4126
	Q10M-112		IGHV5-157*02_S3096	IGHD3-15*01	IGHJ1-1*01	AKSPVYEDDYGYSYTGAYFDF	5.8	7.1	21	IGLV1-NL_9*01_S1120_kim	IGLJ1*01
	Q10M-122		IGHV4-NL_1*01_S8621	IGHD3-15*01	IGHJ1-1*01	ATTRYDEDDYGYYNLEYFDV	6.5	10.3	20	IGLV6-112*01_kim	IGLJ3*01
	Q10M-137		IGHV5-157*02_S3096	IGHD3-15*01	IGHJ5-4*02	AKHDVYEDDYGSSSASWFDV	8.5	15.3	20	IGLV1-NL_9*01_S1120_kim	IGLJ3*01
	Q10M-209		IGHV5-157*02_S3096	IGHD3-15*01	IGHJ4-3*01_S6302	ARAGEYEDEDGSFYTASHFDF	9.5	18.4	21	IGLV1-NL_9*01_S1120_kim	IGLJ2A*01
Q12	Q12BBM-069	Q12BBM-069	IGHV4-174*01	IGHD3-15*01	IGHJ2*01	TKDAIRGYEDEFDGYYTLNDFYFDV	10.8	21.4	25	IGKV1-25*01_kim	IGKJ4*01
	Q12M-165		IGHV4-174*01	IGHD3-15*01	IGHJ2*01	AKEAVRGYEDEFDGYYTLNDWYFDQ	14.5	21.4	25	IGKV1-25*01_kim	IGKJ4*01
	Q12M-061		IGHV4-174*01	IGHD3-15*01	IGHJ2*01	AKEAVRDYEDEFDGYYTLNDFYFDV	13.9	22.4	25	IGKV1-25*01_kim	IGKJ4*01
	Q12QBM-007	Q12QBM-007	IGHV4-117*01	IGHD3-15*01	IGHJ2*01	AKEAIRGYEDEFDGYYTLNDYYYDV	7.2	15.5	25	IGKV1-25*01_kim	IGKJ4*01
	Q12BBM-023		IGHV4-117*01	IGHD3-15*01	IGHJ2*01	AKEAIRGYEDEFDGYYTLNDWYFDV	9.1	17.3	25	IGKV1-25*01_kim	IGKJ4*01
	Q12BBM-068		IGHV4-117*01	IGHD3-15*01	IGHJ2*01	SKEAIRGYEDEFDGYYTLNDWYFDV	9.8	17.3	25	IGKV1-25*01_kim	IGKJ4*01
	Q12M-031		IGHV4-117*01	IGHD3-15*01	IGHJ2*01	AKEAIRGYEDDFDGYYTLNDWYFDL	11.1	19.4	25	IGKV1-25*01_kim	IGKJ4*01
	Q12M-032		IGHV4-117*01	IGHD3-15*01	IGHJ2*01	AKEALRGYEDEFDGYYTLNDFYFDV	7.8	14.3	25	IGKV1-25*01_kim	IGKJ4*01
	Q12M-035		IGHV4-117*01	IGHD3-15*01	IGHJ4-3*01	AGDTEYYEDDGYSYIGLDYFHS	7.8	17.3	22	IGKV1-NL_15*03_S6916_kim	IGKJ4*01
	Q12M-131	Q12M-131	IGHV4-117*01	IGHD3-15*01	IGHJ2*01	AKDALGGYEDDYGYYTVNDFYFDV	7.5	12.4	24	IGKV1-25*01_kim	IGKJ4*01
	Q12M-158		IGHV4-117*01	IGHD3-15*01	IGHJ2*01	AKDALGGYEDDYGYYTVNDFYFDV	7.9	12.4	24	IGKV1-25*01_kim	IGKJ4*01
	Q12M-197		IGHV4-117*01	IGHD3-15*01	IGHJ2*01	AKDALGGYEDDYGYYTVNDFYFDV	9.2	14.4	24	IGKV1-25*01_kim	IGKJ4*01
	Q12M-190	Q12M-190	IGHV4-117*01	IGHD3-15*01	IGHJ2*01_S5087	ARDGGAYYEEDDDFYYLGDWFFDI	5.7	9.2	24	IGKV1-25*01_kim	IGKJ4*01
	Q12M-283		IGHV4-117*01	IGHD3-15*01	IGHJ2*01_S5087	ARDGGAYHEDDYGYYYTGDWYFDI	4.1	10.2	24	IGKV1-25*01_kim	IGKJ4*01
	Q12BBM-045	Q12BBM-045	IGHV3-50*01	IGHD3-15*01	IGHJ3-2*01	ASLDYEDDYGYYYTDGGVFDL	5.8	11.3	21	IGKV1-NL_11*02_S1805_kim	IGKJ1*01
	Q12QBM-034		IGHV3-50*01	IGHD3-15*01	IGHJ3-2*01	ASLDYEDDYGYYYTDGGVFDF	8.2	16.5	21	IGKV1-NL_11*02_S1805_kim	IGKJ1*01
	Q12BBM-056		IGHV3-50*01	IGHD3-15*01	IGHJ3-2*01	ACLDYEDDYGYYYTDGGVFDL	12.1	17.6	21	IGKV1-NL_11*02_S1805_kim	IGKJ1*01
	Q12BBM-065		IGHV3-50*01	IGHD3-15*01	IGHJ3-2*01	ASLDYEDDYGYYYTDGGVFDL	6.5	13.4	21	IGKV1-NL_11*02_S1805_kim	IGKJ1*01
	Q12BBM-085		IGHV3-50*01	IGHD3-15*01	IGHJ3-2*01	ASLDYEDDYGYYYTDGGVFDF	6.5	8.2	21	IGKV1-NL_11*02_S1805_kim	IGKJ1*01
	Q1216M-031	Q1216M-031	IGHV3-122*01	IGHD3-15*01	IGHJ5-5*01	ATTPTYSEDGYGNSQVDSLDV	3.8	6.2	21	IGLV1-85*01_S8291_kim	IGLJ2A*01
	Q12B16M-007		IGHV3-122*01	IGHD3-15*01	IGHJ5-5*01	ATTPTYSEDGYGNSQVDSLDV	3.8	7.2	21	IGLV1-85*01_S8291_kim	IGLJ2A*01
	Q12BBM-042	Q12BBM-042	IGHV3-NL_17*01_S4250	IGHD3-15*01	IGHJ4-3*01	TTPDYVDDYGYFHTHLGGDY	4.7	6	20	IGKV2-72*01_kim	IGKJ3*01
	Q12BBM-076		IGHV3-NL_17*01_S4250	IGHD3-15*01	IGHJ4-3*01	TAADFEDDYGDFYTHLGGDY	4	7	20	IGKV2-72*01_kim	IGKJ3*01
	Q12BBM-022	Q12BBM-022	IGHV4-NL_16*01_S3062	IGHD3-15*01	IGHJ5-5*01	ASEDYEDDFGFYYNVGSLDV	7.7	15.2	20	IGKV1-43*02_kim	IGKJ1*01
	Q12BBM-026		IGHV4-NL_16*01_S3062	IGHD3-15*01	IGHJ5-5*01	ASEDYEDDFGVFYDVGSVDV	11.7	18.2	20	IGKV1-43*02_kim	IGKJ1*01
	Q12BBM-044		IGHV4-NL_16*01_S3062	IGHD3-15*01	IGHJ5-5*01	ASEDYEDDFGVFYDVGSVDV	9.7	19.2	20	IGKV1-43*02_kim	IGKJ1*01
	Q12BBM-043		IGHV3-128*01_S1128	IGHD3-15*01	IGHJ4-3*01	TTDDFEDDYGSFYNVGGTDY	5	7	20	IGKV2-72*01_kim	IGKJ1*01

Supplementary Table 1. Sort statistics and antibody genetics

(a) Sort statistics from NHPs Q7, Q9, Q10, and Q12. Sampling time points, probes used for sorting, numbers of stained PBMCs, sorted cells, productive paired chains, and cloned mAbs are shown. Clonally related mAbs are indicated with identical symbols. (b) Genetic features of the expressed mAbs from NHPs Q7, Q9, Q10, and Q12. The mAbs highlighted in red are shown in Figure 3b.

Supplementary Table 2. X-ray Data collection and refinement statistics (molecular replacement)

	Q9M-023	Q10M-055	Q12BQM-007	Q12BBM-069
**Data collection**				
Space group	P 21 21 21	P 21 21 21	P 21 21 21	P 21 21 21
Cell dimensions				
a, *b*, *c* (Å)	61.7 73.0 132.5	50.7 74.5 135.6	72.9 89.1 89.8	56.4 72.5 175.2
α, β, γ (°)	90 90 90	90 90 90	90 90 90	90 90 90
Resolution (Å)	33.13 - 2.34 (2.38 - 2.34)	33.73 - 1.63 (1.66 - 1.63)	35.14 - 1.54 (1.57 - 1.54)	33.50 - 1.79 (1.82 - 1.79)
R_{sym} or R_{merge}	0.22 (2.5)	0.08 (2.5)	0.10 (1.4)	0.12 (2.4)
$I / \sigma I$	8.5 (1.2)	15.5 (1.0)	13.0 (1.0)	13.9 (1.3)
Completeness (%)	99.9 (99.5)	100.0 (100.0)	98.2 (83.3)	99.6 (99.2)
Redundancy	13.7 (14.1)	13.6 (13.9)	10.3 (5.2)	13.8 (13.7)
**Refinement**				
Resolution (Å)	33.13 - 2.34	33.73 - 1.63	35.14 - 1.54	33.50 - 1.79
No. reflections	26,094 (1289)	65,008 (3204)	84,729 (4039)	68,711 (3407)
R_{work} / R_{free}	0.23 / 0.27	0.22 / 0.25	0.19 / 0.21	0.21 / 0.24
No. atoms	3367	3472	3658	3576
Protein	3347	3238	3372	3355
Ligand/ion	-	-	-	-
Water	20	234	286	221
B-factors				
Protein	53	31	21	34
Ligand/ion	-	-	-	-
Water	45	35	29	38
R.m.s. deviations				
Bond lengths (Å)	0.002	0.012	0.013	0.013
Bond angles (°)	0.5	1.2	1.3	1.3

*Values in parentheses are for highest-resolution shell.

Supplementary Table 3. CryoEM data collection, refinement and validation statistics

	Q10M-055 Fab + Q23 NFL TD CC3+ (EMD-72009) (PDB 9PY5)	Q12QBM-007 Fab + BG505 NFL TD CC3+ (EMD-72031) (PDB 9PYD)	Q12BBM-069 Fab + BG505 NFL TD CC3+ (EMD-72035) (PDB 9PYK)	Q9M-023 Fab + BG505 NFL TD CC3+ (EMD-72033) (PDB 9PYH)	Q7M-675 Fab + WITO NFL TD CC3+ (EMD-74449)
**Data collection and processing**					
Microscope	TFS Glacios 2	TFS Glacios 2	TFS Glacios 2	TFS Glacios 2	TFS Glacios 2
Voltage (keV)	200	200	200	200	200
Camera	TFS Falcon 4i	TFS Falcon 4i	TFS Falcon 4i	TFS Falcon 4i	TFS Falcon 4i
Collection mode	Counting	Counting	Counting	Counting	Counting
Magnification	190,000x	190,000x	190,000x	190,000x	190,000x
Pixel size at detector (Å)	0.718	0.718	0.718	0.718	0.718
Total electron exposure (e^{-}/Å^2)	45.2	44.9	45.0	45.0	45.1
Exposure rate (e^{-}/pixel/sec)	7.56	6.68	7.30	7.84	7.78
Number of EER frames	40	40	40	40	40
Defocus range (µm)	-0.8 to -1.7	-0.8 to -1.7	-0.8 to -1.8	-0.8 to -1.8	-0.8 to -1.8
Automation software	EPU	EPU	EPU	EPU	EPU
Micrographs collected (no.)	7,036	7,003	7,001	7,021	10,912
Micrographs used (no.)	4,663	5,551	6,207	6,766	10,169
Initial particle images (no.)	425,148	947,031	790,420	645,545	1,708,227
Final particle images (no.)	24,541	81,859	72,419	131,528	24,436
Map pixel size (Å)	1.034	1.005	1.034	0.718	0.718
Symmetry	C1	C1	C1	C1	C1
Map resolution (masked/unmasked Å)	3.5/7.1	3.3/4.2	3.3/4.1	3.3/3.8	4.3/7.3
FSC threshold	0.143	0.143	0.143	0.143	0.143
Map sharpening *B* factor (Å^2)	-45	-62	-66	-87	-92
Map resolution range (Å)[#]	3.0-5.0	2.5-4.5	2.5-4.5	2.5-4.0	*n/a*
**Refinement**					
Initial model source	AlphaFold3	AlphaFold3 & PDB 6V0R	AlphaFold3	AlphaFold3	*n/a*
Refinement package	Phenix RSR	Phenix RSR	Phenix RSR	Phenix RSR	*n/a*
Model resolution (Å)	3.8	3.5	3.5	3.4	*n/a*
FSC threshold	0.5	0.5	0.5	0.5	*n/a*
EMRinger score	2.05	2.16	2.40	3.13	*n/a*
CC (mask)	0.78	0.79	0.81	0.82	*n/a*
Model composition					
Non-hydrogen atoms	16,706	15,794	15,785	15,625	*n/a*
Protein residues	1,966	1,896	1,883	1,857	*n/a*
Ligands	84	65	71	73	*n/a*
Mean B factors (Å^2)					
Protein	101	66	49	64	*n/a*
Ligand	108	79	71	81	*n/a*
R.m.s. deviations					
Bond lengths (Å)	0.005	0.007	0.006	0.005	*n/a*
Bond angles (°)	0.999	1.279	1.112	0.879	*n/a*
Validation					
MolProbity score	1.12	1.34	1.25	1.16	*n/a*
Clashscore	1.82	2.66	1.44	1.13	*n/a*
Poor rotamers (%)	0.81	0.42	0.54	0.00	*n/a*
Ramachandran plot					
Favored (%)	96.99	95.82	94.55	95.19	*n/a*
Allowed (%)	3.01	4.18	5.45	4.81	*n/a*
Disallowed (%)	0.00	0.00	0.00	0.00	*n/a*
Cβ outliers (%)	0.00	0.00	0.00	0.00	*n/a*
CaBLAM outliers (%)	1.48	2.80	2.18	3.24	*n/a*

[#]modeled regions

Supplementary Table 4. Antibody heavy and light chain Env contacts and SHM

Contacts colored: Q9M-023 / Q10M-055 / Q12BBM-069 / Q12QBM-007

bold with * are SHM
SHM annotated only to residue 94

	Q9M-023 HC Kabat #	Q9M-023 HC aa	Q10M-055 HC Kabat #	Q10M-055 HC aa	Q12BBM-069 HC Kabat #	Q12BBM-069 HC aa	Q12QBM-007 HC Kabat #	Q12QBM-007 HC aa	EPITOPE
	1	Q	1	Q	1	Q	1	Q	
	2	L	2	L	2	V*	2	V*	
	3	Q	3	R*	3	H*	3	Q	
	4	L	4	L	4	L	4	L	
	5	Q	5	Q	5	Q	5	Q	
	6	E	6	E	6	E	6	E	
	7	S	7	S	7	S	7	S	
	8	G	8	G	8	G	8	G	
	9	P	9	P	9	P	9	P	
	10	G	10	G	10	G	10	G	
	11	L	11	L	11	L	11	L	
	12	V	12	V	12	V	12	V	
	13	K	13	E*	13	K	13	K	
	14	P	14	P	14	P	14	P	
	15	S	15	L	15	S	15	S	
	16	E	16	E	16	E	16	E	
	17	T	17	T	17	T	17	T	
	18	L	18	L	18	L	18	L	
	19	S	19	S	19	S*	19	S	
	20	L	20	L	20	L	20	L	
	21	T	21	T	21	T	21	T	
	22	C	22	C	22	C	22	C	
	23	T*	23	A	23	A	23	D*	
	24	V	24	V	24	V	24	V	
	25	S	25	S	25	S	25	S	
	26	G	26	G	26	G	26	G	
	27	V*	27	G	27	A	27	A*	
	28	F*	28	F*	28	S	28	S	
	29	I	29	V*	29	I	29	I	
HCDR1	30	D*	30	S	30	N*	30	S	V2b V2
	31	T*	31	S	31	Y*	31	S	
	32	N	32	D*	32	N	32	N	
	33	Y	33	Y	33	Y	33	Y	
	34	W	34	W	34	W	34	W	
	35	S	35	Y*	35	N*	35	N*	
	36	W	36	W	36	W	36	W	
	37	I	37	I	37	I	37	I	
	38	R	38	R	38	R	38	R	
	39	Q	39	Q	39	Q	39	Q	
	40	S*	40	P	40	A	40	T*	
	41	P	41	P	41	P	41	P	
	42	G	42	G	42	G	42	G	
	43	K	43	K	43	K	43	K	
	44	G	44	G	44	G	44	G	
	45	L	45	L	45	L	45	L	
	46	E	46	E	46	E	46	E	
	47	W	47	C*	47	W	47	W	
	48	I	48	I	48	I	48	I	
	49	G	49	G	49	G	49	G	
	50	R	50	R	50	R	50	R	
	51	I	51	I	51	L*	51	L*	
	52	S	52	S	52	D*	52	D*	
	52a	G	52a	G	52a	G	52a	G	
HCDR2	53	D*	53	R*	53	N*	53	S	
	54	G	54	H*	54	S*	54	G	
	55	G	55	G	55	G	55	G	
	56	D*	56	N*	56	S	56	S	V2 V3
	57	T	57	T	57	T	57	A*	
	58	D	58	D	58	D	58	D	
	59	N*	59	Y	59	L*	59	Y	
	60	N	60	S*	60	N	60	N	
	61	P	61	P	61	P	61	P	
	62	S	62	S	62	S	62	S	
	63	L	63	L	63	L	63	L	
	64	K	64	K	64	R*	64	R*	
	65	S	65	S	65	S	65	S	
	66	R	66	R	66	R	66	R	
	67	V	67	V	67	V	67	V	
	68	T	68	T	68	T	68	T	
	69	I	69	I	69	F*	69	I	
	70	S	70	S	70	S	70	S	
	71	I*	71	T	71	T*	71	T	
	72	D	72	D	72	D	72	D	
	73	T	73	T	73	T	73	A*	
	74	A*	74	S	74	S	74	S	
	75	K	75	K	75	K	75	K	
	76	N	76	N	76	D*	76	D*	
	77	Q	77	Q	77	H*	77	Q	
	78	F	78	L*	78	F	78	V*	
	79	S	79	S	79	S	79	S	
	80	L	80	L	80	L	80	L	
	81	R*	81	N*	81	R*	81	N*	
	82	L	82	L	82	L	82	L	
	82a	I*	82a	K*	82a	T*	82a	R*	
	82b	S	82b	S	82b	S	82b	S	
	82c	V	82c	V	82c	V	82c	V	
	83	T	83	T	83	T	83	T	
	84	A	84	T*	84	A	84	A	
	85	A	85	A	85	A	85	A	
	86	D	86	D	86	D	86	D	
	87	T	87	T	87	T	87	T	
	88	A	88	A	88	A	88	A	
	89	V	89	V	89	V	89	V	
	90	Y	90	Y	90	Y	90	Y	
	91	Y	91	Y	91	F*	91	F*	
	92	C	92	C	92	C	92	C	
	93	A	93	A	93	T*	93	A	
	94	R	94	S*	94	K*	94	K	
	95	P	95	D	95	D	95	E	
	96	V	96	L	96	A	96	A	
	97	R	97	Y	97	I	97	I	
	98	A	98	Y	98	R	98	R	
	99	D	99	E	99	G	99	G	
	100	H	100	D	100	Y	100	Y	
	100a	E	100a	D	100a	E	100a	E	
HCDR3	100b	D	100b	Y	100b	D	100b	D	
	100c	D	100c	G	100c	E	100c	E	
	100d	D	100d	Y	100d	F	100d	F	
	100e	G	100e	S	100e	D	100e	D	
	100f	Y	100f	Y	100f	G	100f	G	V2 N160 glycan
	100g	Y	100g	E	100g	Y	100g	Y	
	100h	Y	100h	G	100h	Y	100h	Y	
	100i	S	100i	L	100i	T	100i	T	
	100j	Y	100j	D	100j	L	100j	L	
	100k	D	100k	V	100k	N	100k	N	
	100l	D	100l	F	100l	D	100l	D	
	100m	A	101	D	100m	F	100m	Y	
	100n	F	102	F	100n	Y	100n	Y	
	101	D	103	W	100o	F	100o	Y	
	102	L	104	G	101	D	101	D	
	103	W	105	Q	102	V	102	V	
	104	G	106	G	103	W	103	W	
	105	Q	107	L	104	G	104	G	
	106	G	108	R	105	P	105	P	
	107	L	109	V	106	G	106	G	
	108	R	110	T	107	I	107	T	
	109	V	111	V	108	P	108	P	
	110	T	112	S	109	V	109	I	
	111	V	113	S	110	T	110	T	
	112	S			111	I	111	I	
	113	S			112	S	112	S	
					113	S	113	S	

	Q9M-023 LC Kabat #	Q9M-023 LC aa	Q10M-055 LC Kabat #	Q10M-055 LC aa	Q12BBM-069 LC Kabat #	Q12BBM-069 LC aa	Q12QBM-007 LC Kabat #	Q12QBM-007 LC aa	EPITOPE
	1	D	1	E	1	D	1	D	
	2	I	2	I	2	I	2	I	
	3	Q	3	V	3	Q	3	Q	
	4	M	4	M	4	M	4	M	
	5	T	5	T	5	T	5	T	
	6	Q	6	Q	6	Q	6	Q	
	7	S	7	S	7	S	7	S	
	8	P	8	P	8	P	8	P	
	9	S	9	A	9	S	9	P*	
	10	S	10	T	10	S	10	S	
	11	L	11	L	11	L	11	L	
	12	A*	12	S	12	S	12	S	
	13	A	13	L	13	A	13	A	
	14	S	14	S	14	S	14	S	
	15	V	15	P	15	V	15	V	
	16	G	16	G	16	G	16	G	
	17	D	17	E	17	D	17	D	
	18	R	18	R	18	R	18	T*	
	19	V	19	A	19	V	19	V	
	20	T	20	T	20	S*	20	T	
	21	I	21	L	21	I	21	I	
	22	T	22	S	22	T	22	T	
	23	C	23	C	23	C	23	C	
	24	R	24	R	24	R	24	R	
	25	A	25	A	25	T*	25	A	
	26	S	26	S	26	S	26	S	
	27	Q	27	Q	27	Q	27	H*	
	28	G	28	S	28	D*	28	D*	
	29	I	29	V	29	I	29	I	N160 glycan
LCDR1	30	S	30	S	30	S	30	S	
	31	G*	31	Y*	31	S	31	S	
	32	W	32	N*	32	Y	32	F*	
	33	L	33	L	33	L	33	L	
	34	A	34	A	34	A	34	A	
	35	W	35	W	35	W	35	W	
	36	Y	36	Y	36	Y	36	Y	
	37	Q	37	Q	37	Q	37	Q	
	38	Q	38	Q	38	Q	38	Q	
	39	K	39	K	39	K	39	K	
	40	P	40	P	40	P	40	P	
	41	G	41	G	41	G	41	G	
	42	T*	42	Q	42	K	42	K	
	43	A	43	A	43	A	43	A	
	44	P	44	P	44	P	44	P	
	45	R*	45	R	45	K	45	K	
	46	L	46	L	46	L	46	L	
	47	L	47	L	47	L	47	L	
	48	I	48	V*	48	I	48	I	
	49	H*	49	Y	49	Y	49	Y	
	50	K	50	G	50	K	50	K	
LCDR2	51	A	51	A	51	A	51	A	
	52	S	52	S	52	S	52	S	
	53	T	53	S	53	A*	53	A*	
	54	deletion	54	R	54	L	54	L	
	55	L	55	A	55	Q	55	Q	
	56	N*	56	T	56	D*	56	D*	
	57	G	57	G	57	G	57	G	
	58	V	58	I	58	V	58	V	N160 glycan
	59	P	59	P	59	P	59	P	
	60	S	60	D	60	S	60	S	
	61	R	61	R	61	R	61	R	
	62	F	62	F	62	F	62	F	
	63	S	63	S	63	S	63	S	
	64	G	64	G	64	G	64	G	
	65	S	65	S	65	S	65	S	
	66	G	66	G	66	G	66	G	
	67	Y*	67	S	67	S	67	S	
	68	G	68	G	68	G	68	G	
	69	T	69	T	69	T	69	T	
	70	D	70	E	70	E*	70	D	
	71	F	71	F	71	F	71	F	
	72	T	72	T	72	T	72	T	
	73	L	73	L	73	L	73	L	
	74	T	74	T	74	T	74	T	
	75	I	75	I	75	I	75	I	
	76	S	76	T*	76	S	76	S	
	77	S	77	S	77	S	77	R*	
	78	L	78	L	78	L	78	L	
	79	Q	79	E	79	Q	79	Q	
	80	P	80	P	80	P	80	P	
	81	E	81	E	81	E	81	E	
	82	D	82	D	82	D	82	D	
	83	F	83	F	83	F	83	F	
	84	A	84	A	84	A	84	A	
	85	T	85	F*	85	T	85	I*	
	86	Y	86	Y	86	Y	86	Y	
	87	Y	87	F*	87	Y	87	Y	
	88	C	88	C	88	C	88	C	
	89	Q	89	Q	89	Q	89	Q	
	90	Q	90	Q	90	Q	90	Q	
	91	Y	91	Y	91	H	91	H	
	92	K*	92	I*	92	N	92	N	
LCDR3	93	S	93	H*	93	Y	93	Y	V1 V2
	94	V*	94	W	94	I	94	I	
	95	P	95	P	95	P	95	P	
	96	Y	96	T	96	L	96	L	
	97	S	97	F	97	T	97	T	
	98	F	98	G	98	F	98	F	
	99	G	99	Q	99	G	99	G	
	100	Q	100	G	100	G	100	G	
	101	G	101	T	101	G	101	G	
	102	T	102	K	102	T	102	T	
	103	K	103	V	103	N	103	N	
	104	V	104	E	104	V	104	V	
	105	E	105	I	105	E	105	E	
	106	I	106	K	106	I	106	I	
	107	K			107	K	107	K	

Supplementary Table 4. Antibody heavy and light chains Env contacts and SHM
Antibody residues contacting Env are highlighted in color with Env region denoted in grey. Antibody residues resulting from SHM are marked with an asterisk in bold font. SHM only annotated to residue 94.
