# Supplementary material for: Vaccination generates broadly cross-neutralizing antibodies to the HIV Env apex
Source: Nature. 2026 Apr 29;654(8119):777–85. doi: 10.1038/s41586-026-10429-3 (PMC13275315; doi:10.1038/s41586-026-10429-3)
Supplement: Supplementary file 2 — Reporting Summary [file 41586_2026_10429_MOESM2_ESM.pdf]

nature portfolio

Corresponding author(s): Richart T. Wyatt

Last updated by author(s): March 05, 2026

Reporting Summary

Nature Portfolio wishes to improve the reproducibility of the work that we publish. This form provides structure for consistency and transparency in reporting. For further information on Nature Portfolio policies, see our Editorial Policies and the Editorial Policy Checklist.

Please do not complete any field with "not applicable" or n/a. Refer to the help text for what text to use if an item is not relevant to your study. For final submission: please carefully check your responses for accuracy; you will not be able to make changes later.

Statistics

For all statistical analyses, confirm that the following items are present in the figure legend, table legend, main text, or Methods section.

n/a	Confirmed	
☐	☒	The exact sample size (*n*) for each experimental group/condition, given as a discrete number and unit of measurement
☐	☒	A statement on whether measurements were taken from distinct samples or whether the same sample was measured repeatedly The statistical
☒	☐	test(s) used AND whether they are one- or two-sided<br>*Only common tests should be described solely by name; describe more complex techniques in the Methods section.*
☒	☐	A description of all covariates tested
☒	☐	A description of any assumptions or corrections, such as tests of normality and adjustment for multiple comparisons
☒	☐	A full description of the statistical parameters including central tendency (e.g. means) or other basic estimates (e.g. regression coefficient) AND variation (e.g. standard deviation) or associated estimates of uncertainty (e.g. confidence intervals)
☒	☐	For null hypothesis testing, the test statistic (e.g. *F*, *t*, *r*) with confidence intervals, effect sizes, degrees of freedom and *P* value noted<br>*Give P values as exact values whenever suitable.*
☒	☐	For Bayesian analysis, information on the choice of priors and Markov chain Monte Carlo settings
☒	☐	For hierarchical and complex designs, identification of the appropriate level for tests and full reporting of outcomes ☐
☒		Estimates of effect sizes (e.g. Cohen's *d*, Pearson's *r*), indicating how they were calculated

Our web collection on statistics for biologists contains articles on many of the points above.

Software and code

Policy information about availability of computer code Data

collection	Crystallography: Data were collected at the NSLS-II AMX beamline using the automated pipeline. Data processing was performed with autoPROC (Global Phasing Ltd.) employing XDS (build 20241002), POINTLESS (v1.12.14), AIMLESS (v0.7.9), TRUNCATE, and CCP4 (v8.0.010). CryoEM data: EPU (3.13.0)<br>Neutralization and ELISA assays: Gen5 3.11 BLI:Octet Data<br>Aquisition 11.1.3.25<br>DSC: MicroCal VP- Capillary DSC softaware 2.0
Data analysis	Data processing was performed with autoPROC (Global Phasing Ltd.) employing XDS (build 20241002), POINTLESS (v1.12.14), AIMLESS (v0.7.9), TRUNCATE, and CCP4 (v8.0.010). Model building, refinement, validation, and figure preparation were carried out using Coot (v0.9.8.95), Phenix (v1.21.2-5419), and PyMOL (v2.5.4). Relion (4.0)<br>UCSF ChimeraX (1.10.1)<br>cryoSPARC (v4.7.1) Coot<br>(0.9.8)<br>Phenix (1.21.2)<br>IgDiscover (v1.0.4) Pear<br>(v0.9.6) FlowJo<br>(v10.10.0) Python 3<br>R version 4.5.1

Snapgene 8.1
GraphPad Prism 10.6.0 Data
Analysis Forte Bio 11.1 Origen v7
Excel 16.105.1

For manuscripts utilizing custom algorithms or software that are central to the research but not yet described in published literature, software must be made available to editors and reviewers. We strongly encourage code deposition in a community repository (e.g. GitHub). See the Nature Portfolio guidelines for submitting code & software for further information.

Data

Policy information about availability of data

All manuscripts must include a data availability statement. This statement should provide the following information, where applicable:

- Accession codes, unique identifiers, or web links for publicly available datasets
- A description of any restrictions on data availability
- For clinical datasets or third party data, please ensure that the statement adheres to our policy

The HC VDJ and LC VJ sequences of the Env-specific mAbs have been deposited in GenBank under the codes: For the Heavy Chains: PX281429-PX281473, PX717267-PX717279; for the Kappa Chains: PX281474-PX281512, PX717254-PX717264; and for the Lambda Chains: PX281513-PX281518, PX717265-PX717266. IgM, IgK and IgL repertoire data are available from ENA under the codes: Q7 IGM ERR16022197, Q7 IGK ERR16022198, Q7 IGL ERR16022199, Q9 IGM ERR15498913, Q9 IGK ERR15498914, Q9 IGL ERR15498915, Q10 IGM ERR15498916, Q10 IGK ERR15498917, Q10 IGL ERR15498918, Q12 IGM ERR15498919, Q12 IGK ERR15498920, Q12 IGL ERR15498921. The IgDiscover software can be found at https://gkhlab.gitlab.io/igdiscover22/. CryoEM maps have been deposited in the Electron Microscopy Data Bank (EMDB) under accession codes EMD-72009, EMD-72031, EMD-72033, EMD-72035 and EMD-74449, and cryoEM models have been deposited in the Protein Data Bank (PDB) under accession codes 9PY5, 9PYD, 9PYK and 9PYH. X-ray crystal structures of the Fabs have been deposited in the PDB under the codes 9PYN, 9PYY, 9PZ2 and 9PZ3. Representative negative stain EMPEM maps have been deposited into the Electron Microscopy Data Bank under accession codes EMD-72735, EMD-72736, EMD-72737, EMD-72738 and EMD-72739. All deposited data is publicly available.

Human research participants

Policy information about studies involving human research participants and Sex and Gender in Research.

Reporting on sex and gender	N/A
Population characteristics	N/A
Recruitment	N/A
Ethics oversight	N/A

Note that full information on the approval of the study protocol must also be provided in the manuscript.

Field-specific reporting

Please select the one below that is the best fit for your research. If you are not sure, read the appropriate sections before making your selection.

☒ Life sciences ☐ Behavioural & social sciences ☐ Ecological, evolutionary & environmental sciences

For a reference copy of the document with all sections, see nature.com/documents/nr-reporting-summary-flat.pdf

Life sciences study design

All studies must disclose on these points even when the disclosure is negative.

Sample size

For the macaque immunization experiment, we established sample size primarily by extensive historical experience in similar vaccine and challenge studies. We have taken into consideration the "three Rs" principle that guides the humane use of animals in scientific research, and in particular the 2nd "R" (Reduction). The minimum number of animals was used to achieve the study's objective while minimizing the use of highly sentient species.

Data exclusions

No data exclusion.

Replication

Neutralization activity of the serum IgG and the monoclonal antibodies isolated in this study was assessed twice in independent experiments with similar results. Bio-layer-Light interferometry experiments were repeated twice and a representative experiment is reported.
Duplication of EMPEM sample imaging is not feasible due to the high costs associated with image collection and data processing. Nonetheless, we follow standard operating procedures for EMPEM, which includes, per sample, a minimum of 100,000 individual observations of immune complexes (trimers and trimers in complex with polyclonal Fabs) analyzed by 2D classification, and a minimum of 50,000 observations ("particles") subjected to 3D classification. The final stack of particles used to reconstruct a 3D map are also analyzed by 2D classification to

ensure that any Fab density visible in 3D maps is corroborated by the experimental data (2D particles). All micrographs from data collection are archived and available for independent re-processing.

Randomization	N/A
Blinding	N/A

Reporting for specific materials, systems and methods

We require information from authors about some types of materials, experimental systems and methods used in many studies. Here, indicate whether each material, system or method listed is relevant to your study. If you are not sure if a list item applies to your research, read the appropriate section before selecting a response.

Materials & experimental systems

n/a	Involved in the study	
☐	☒	Antibodies Eukaryotic
☐	☒	cell lines
☒	☐	Palaeontology and archaeology Animals
☐	☒	and other organisms Clinical data
☒	☐	Dual use research of concern
☒	☐	

Methods

n/a	Involved in the study	
☒	☐	ChIP-seq
☐	☒	Flow cytometry
☒	☐	MRI-based neuroimaging

Antibodies

Antibodies used	Peroxidase AffiniPure™ Goat Anti-Human IgG (H+L) Jackson Lab Cat#109-035-088 BD Pharmingen™ FITC Mouse Anti-Human CD3ε BD Bioscience Cat#556611 BD Pharmingen™ FITC Mouse Anti-Human CD14 BD Bioscience Cat#557153 BD Pharmingen™ PE-Cy™7 Mouse Anti-Human CD27 BD Bioscience Cat#560609 BD Horizon™ PE-CF594 Mouse Anti-Human IgG BD Bioscience Cat#562538 BD Horizon™ BV421 Mouse Anti-Human CD20 BD Bioscience Cat#562873 BD Horizon™ PerCP-Cy5.5 Mouse Anti-Human CD20 BD Bioscience Cat#560736 NHP Fc Block eBioscience Cat#14-9165-42 PG9, CH01, RHA1, VRC01, VRC26, BG18, 447-52D, PGT145 and F105 (Wyatt Laboratory)
Validation	Antibodies that were commercially obtained (Jackson Laboratories, BD Bioscience, eBioscience) were not validated internally as we relied on quality control checks and reports from the manufacturer. The mAbs PG9, CH01, RHA1, VRC01, VRC26, BG18, 447-52D, PGT145 and F105 were validated internally by binding to HIV Env antigens via ELISA and Bio-layer interferometry (BLI).

Eukaryotic cell lines

Policy information about cell lines and Sex and Gender in Research Cell line

source(s)	Human: FreeStyle 293F cells Invitrogen Cat#R79007 Human: TZM-bl cells NIH AIDS Reagent Program Cat#8129 Human: 293T cells ATCC Cat#CRL-3216 Human: Expi293F cells Thermo Fisher Scientific Cat#A14527
Authentication	No internal authentication was performed
Mycoplasma contamination	All cell lines tested negative for mycoplasma contamination. Screened using the MycoAlertTM Mycoplasma Detection Kit (Lonza, #LT07-118) or tested with MycoStrip Mycoplasma Detection Kit, Sourced from InvivoGen (Cat#: rep-mys-10)
Commonly misidentified lines (See ICLAC register)	No commonly misidentified lines were used in the study

Animals and other research organisms

Policy information about studies involving animals; ARRIVE guidelines recommended for reporting animal research, and Sex and Gender in Research

Laboratory animals	Twelve adult Indian-origin rhesus macaques (Macaca mulatta) (RM) were housed at the Emory National Primate Research Center (ENPRC) and maintained in accordance with NIH guidelines. Rhesus macaques were male and female, an age range of 3-4 years old at the start of the study with an average weight of 4.8 kgs. Animals were grouped to distribute age and weight as evenly as possible

	between the groups receiving either soluble or liposome-conjugated trimers. Animals were housed in pairs for the duration of the study.
Wild animals	No wild animals were used in this study.
Reporting on sex	The current study includes both female and male animals, and while a perfect balancing of the animal sex was not possible for logistical reasons (i.e., animal availability), the immunization groups included two females and four males, thus indicating that the reported findings do not apply to only one gender.
Field-collected samples	No field collected samples were used in this study.
Ethics oversight	The animal work was approved by the Emory University Institutional Animal Care and Use Committee (IACUC) under protocol 202100136. Animal care facilities are accredited by the U.S. Department of Agriculture (USDA) and the Association for Assessment and Accreditation of Laboratory Animal Care (AAALAC) International.

Note that full information on the approval of the study protocol must also be provided in the manuscript.

Flow Cytometry

Plots

Confirm that:

☒ The axis labels state the marker and fluorochrome used (e.g. CD4-FITC).

☒ The axis scales are clearly visible. Include numbers along axes only for bottom left plot of group (a 'group' is an analysis of identical markers). ☒ All plots are contour plots with outliers or pseudocolor plots.

☒ A numerical value for number of cells or percentage (with statistics) is provided.

Methodology

Sample preparation	Frozen single cell suspension from blood mononuclear cells (PBMCs) from animals Q7, Q9, Q10, and Q12 were thawed at 37° C, washed twice in pre-warmed RPMI 1640 media (HyClone) supplemented with 10% FBS (HyClone) and Penicillin/ Streptomycin (100 IU/ml 100 µg/ml) (Gibco). The cells were washed with PBS (Sigma) and counted using trypan blue exclusion of dead cells by a Countess II cell counter (Thermo Fisher). Cells were suspended in PBS and incubated for 30 minutes at 4°C with Live/Dead Fixable Aqua Dead Cell Stain Kit (Life Technologies) according to the manufacturer's instructions. Cells were washed with FACS buffer (PBS + 1% FBS) and surface stained with the following antibodies: CD3 FITC (clone SP34-2), CD14 FITC (clone M5E2), CD20 BV421 or PerCP-Cy5.5 (clone 2H7), CD27 PE-Cy7 (clone M-T271), IgG PE-CF594<br>(clone G18-145) (all from BD Biosciences). Staining was performed for 30 minutes at 4°C. To produce Env trimer probes for B cell sorting, 10 µg of biotinylated Q23, BG505, and 16055 NFL trimers were conjugated to streptavidin-APC (SA APC) (Invitrogen) or SA BV421 (BioLegend) in five sequential steps, each incubation proceeded for 20 min at 4°C. After washing with FACS buffer, cells were subsequently stained with the fluorescently conjugated NFL trimers. Live CD3-CD14-CD20+CD27<br>+IgG+ENV+ single cells were sorted into 96-well PCR plates (Eppendorf) containing 4 µl/well of ice-cold cell lysis buffer (0.5x PBS, 10 mM DTT and 2 U/µl RNAsin (all from Thermo Fisher)). After sorting the 96-well plates were centrifuged, sealed, and immediately frozen on dry ice and stored at -80°C until use.
Instrument	four-laser FACSAria Fusion cell sorter (Becton Dickinson)
Software	FlowJo Version 10.8.1
Cell population abundance	Purity was determined by specific and relevant staining of markers using flow cytometry. Additionally, single-cell index sorting data confirmed the purity of sorted population.
Gating strategy	PBMCs from animals Q7, Q9, Q10, and Q12 were first gated on lymphocytes based on FSC-A/SSC-A, followed by FSC-A/FSC-H to exclude doublets/select singlets. Dead cells, T cells, and monocytes were excluded using Aqua Live/Dead staining and CD3 CD14 markers, respectively. IgG^+ B cells were defined as $CD20^+IgG^+$. Env-specific memory B cells were identified as $CD27^+Env^+$ (Q23, BG505, 16055) within the IgG^+ B cell population.

☒ Tick this box to confirm that a figure exemplifying the gating strategy is provided in the Supplementary Information.

This checklist template is licensed under a Creative Commons Attribution 4.0 International License, which permits use, sharing, adaptation, distribution and reproduction in any medium or format, as long as you give appropriate credit to the original author(s) and the source, provide a link to the Creative Commons license, and indicate if changes were made. The images or other third party material in this article are included in the article's Creative Commons license, unless indicated otherwise in a credit line to the material. If material is not included in the article's Creative Commons license and your intended use is not permitted by statutory regulation or exceeds the permitted use, you will need to obtain permission directly from the copyright holder. To view a copy of this license, visit http://creativecommons.org/licenses/by/4.0/
