# Supplementary material for: Vaccination generates broadly cross-neutralizing antibodies to the HIV Env apex
Source: Nature. 2026 Apr 29;654(8119):777–85. doi: 10.1038/s41586-026-10429-3 (PMC13275315; doi:10.1038/s41586-026-10429-3)
Supplement: Supplementary file 3 — Peer Review File [file 41586_2026_10429_MOESM3_ESM.pdf]

nature portfolio

Peer Review File

HIV Env apex broadly cross-reactive neutralizing antibodies by vaccination

Corresponding Author: Professor Richard Wyatt

Any redactions in this file are there to maintain patient confidentiality, the confidentiality of unpublished data, or to remove third-party material.

This file contains all reviewer reports in order by version, followed by all author rebuttals in order by version.

Version 1:

Reviewer comments:

Referee #1

(Remarks to the Author)
Wyatt and colleagues report an important finding: vaccination of rhesus macaques (RMs) with multivalent, high avidity, tandem Q23 and ZM233 Env trimers bound to liposomes (followed by additional wild-type Env trimer-liposome boosts) induced V2 apex-targeted broadly-neutralizing serum antibodies (bnAbs) in 6 of 6 monkeys. This result – consistent induction of potent V2 apex ns in serum of outbred animals following immunization – has not been previously achieved by any investigative group despite decades of work and represents a watershed moment in HIV-1 vaccine research. The only other studies that approximate this are those showing that extensive immunizations with fusion peptide conjugated to KLH or tetanus toxin followed by Env trimer SOSIP vaccination elicits broadly-reactive but generally low potency antibody responses in small animals and nonhuman primates.

The Wyatt team isolated bnAb mAbs from monkeys with the greatest neutralization breadth and showed that these largely recapitulate the breadth seen in serum. With these mAbs in hand, they could characterize their immunogenetics and structures and confirm the authenticity of the V2 apex targeted responses. Thus, the authors prove for the first time in an outbred animal model that V2 apex bnAb lineages can be efficiently primed by a protein immunogen and relatively easily affinity-matured to acquire neutralization breadth and potency.

The elegance and impact of this manuscript is in its simplicity: take “special Envs” known for their predilection for binding V2 apex bnAbs and bnAb iGL receptors; enhance dramatically their valency and avidity for bnAb precursors; deliver a lot of them (800 ugm) repetitively (q2wks) over an initial 8 week period to a lot of naïve germline target cells (8 separate immunization sites, two in each of four extremities) and immunize five more times over a two-year period . . . and presto: bnAbs!

A potentially important inference that the authors draw that may impact HIV-1 immunogen design more generally is that a key to their success was the use of near-native-like Envs for prime and boosts as opposed to heavily-engineered Envs. The authors suggest by using wildtype Envs as immunogens and increasing their avidity by multivalency, they prime naïve germline B-cells with receptors that from the outset recognize and bind near-native Envs. As a consequence, the next steps of affinity maturation need only “polish” or affinity mature modestly evolving bnAb receptors to bind still more avidly to wildtype Envs and develop breadth and potency. The authors point out that this is a very different strategy than that pursued by some other leading groups in the HIV-1 vaccine field who prime with heavily engineered Envs that bind particular bnAb iGL receptors well and expand them efficiently but which thus far have been unable to develop neutralization breadth and potency . . . probably because the heavily engineered Env immunogens prime naïve germline B cells that have receptors that “look” like bnAb precursors but which at the atomic level are actually quite different. Wyatt and colleagues don’t have this problem because they prime with near-native Env trimers.

I do have a number of concerns and issues with thi manuscript:

Line 102 – Bold heading reads “Env recognized by a germline apex bnAb UCA.” The authors correctly note that Q23 Env binds to the rhesus V2 apex bnAb RHA1 lineage iGL but this is NOT a UCA. Later in the paragraph refer to enhanced

binding of the Q23 liposome to the human V2 apex bnAb CH01 and to the "unmutated common ancestor or UCA" of the CH01 lineage antibodies. The terms "UCA" and "unmutated common ancestor" mean something very specific: a phylogenetic inference where the V, D and J genes plus the nontemplated sequences between V and D and D and J have all been precisely inferred from deep sequencing of Bmem Ig sequences. This was NEVER done for the CH01 lineage and could never have been done because clinical samples from the CH01 subject were not available to do so. Instead, the Haynes group made an "RUA" inference (reverted unmutated ancestor), which is similar to an iGL but certainly nothing approaching a true UCA inference (see Liao et al., Immunity 38:176, 2013 and other Haynes papers). Indeed, one can find in the scientific literature the CH01 lineage precursor referred to as "UCA," "RUA," and "iGL. With respect to the CH01 lineage precursor, use of the term "UCA" is sloppy, misleading and incorrect. Wyatt and colleagues should correct this throughout the manuscript and in Fig. 1D.

Line 136 – I suppose one could say that Q23 and ZM233 immunizations were given in "tandem" but this seems like an awkward and potentially confusing description. Why not write "sequential"? Also, the authors should make clear that the monkeys were primed with Q23 and boosted with ZM233 and then boosted again and again with other wildtype Envs.

Line130-169 – Figs. 1g and Extended Data Fig 1h, and the text describing these results, are difficult to follow because the viruses in the left-most columns are sometimes the same between the figures, sometimes different, and oftentimes not in the same order top to bottom. Furthermore, the figures are used to highlight the "breadth" of the serum IgG responses but some of these viruses are identical to the immunogens these monkeys received: thus, such responses are autologous, not heterologous. It would be more clear if the authors were to separate spatially in the figures responses to autologous Envs from responses to heterologous Envs and indicate which is which. It would also be helpful if Figs. 1g and Extended Data Fig 1h contained an identical set of heterologous Envs in the same order top to bottom, and it would be even better if the list of viruses was longer and included all of the viruses shown in Fig. 2C at a minimum. In other sections of the text, the same confusing situation arises when the authors show breadth and potency of bnAb mAbs, where for example, in Fig. 2c some of the viruses are autologous to the immunogens, others are heterologous, and the order top to bottom doesn't resemble Figs. 1g or Extended Data Fig 1h. What makes this even more difficult to follow is then the authors indicate that some viruses contain N130 and some don't and it is not always clear from the figures or the legends which is which. It might be helpful as an Extended Data figure to have a single table where every virus that was tested for neutralization sensitivity to either serum IgG or mAbs is listed top to bottom; next to that an indication if it contains N130 or not; next to that if it is part of the immunization sequence or not, and next to that the neut results for both polyclonal IgG and mAb. (I don't feel strongly about this latter suggestion if the authors can otherwise address the lack of clarity.)

Line 171-213 – I found this text and the corresponding Fig. 2a and 2b confusing. The authors state that they recovered 103, 159 and 393 paired H and L chains from monkeys Q9, Q10 and Q12, and these grouped into 70, 106 and 203 clonal lineages. To me, a clonal lineage is defined as a lineage of B cells that have evolved from a single germline UCA B cell that was primed and then expanded. If that is what the authors mean, they should say so. Then, they say that they analyzed these lineages and selected those with D3-15 genes and CDRH3s longer than 20aa and a negatively-charged core motif, leading to the identification of 16, 30 and 58 HC and LC pairs, respectively, with these features. Am I correct to assume this means that from Q9, they found 16 expanded bnAb-like lineages, from Q10 they found 30, and from Q12 they found 58. So, for example, using PBMC samples collected 1-2 years post-initial prime, they found 58 distinct bnAb-like lineages? If so, were these primed by Q23, ZM233, WITO or one of the other three immunogens? Finally the authors say they selected 45 HC and LC pairs from this total of 104 to clone and express as mAbs and to assess their neutralization properties . . . leading to the identification of 9 bnAb mAbs corresponding to 5 discreet lineages shown in Fig. 2b. If all this is the case, it would be helpful to the reader to have this spelled out more clearly and for the authors to offer some perspective on how these 5 discreet bnAb lineages relate to the 104 bnAb-like lineages described above. In essence, what I am asking the authors is what is the priming efficiency of their Q23 liposome? How many V2 apex bnAb-like precursors were primed and expanded after the initial prime and boost and how many of these affinity matured with further immunizations to acquire breadth and potency?

Referee #2

(Remarks to the Author)
Key result: The study demonstrates effective immuno-focusing on the V2 apex of the HIV Env in NHPs, leading to the elicitation of tier 2 HIV-1 neutralizing antibodies targeting this region.
Validity: No apparent flaws.
Originality/significance: The results are highly significant.
The findings are significant. The central concept is compelling, the experiments were conducted in a relevant animal model, and the conclusions are well supported by the experimental data. The elicited breadth of neutralization against heterologous tier 2 viruses that is focused on the V2 Apex of Env, is impressive, but is limited to only a subset of viruses with Envs lacking the N130 glycans. The underlying reasons for the neutralization resistance by a large fraction of N130-expressing viruses are not discussed. Although the authors correctly note that the CDRH3 regions of the elicited anti-Apex antibodies share features with known anti-V2 Apex broadly neutralizing antibodies, the underlying mechanistic reasons for their failure to neutralize N130-expressing viruses but also N130-lacking viruses, remain unclear and are not addressed.
Comments:
1. Lines 86-92: After the first two immunizations, autologous neutralizing antibodies were detected in both liposome- Env and soluble Env–immunized animals, yet only apex-directed antibodies in the liposome group are discussed here. They should mention that such responses were also recorded in the soluble Env immunized animals, but at lower frequences.
2. The IgL form of RHA1, which carries the same CDRH3 as the mature antibody, shows minimal or no binding to Q23NFL

and ZM233 NFL, while the mature antibody binds them with KDs near 10^{-10} (Fig. 1b). This suggests that the CDRH3 alone is insufficient for broad neutralization. Which additional features of V2 apex-directed bnAbs contribute to Env engagement and breadth? Do the elicited NHP antibodies lack these features? Understanding this would clearly inform improved immunogen design.
3. Ext Fig. 2: A substantial fraction of N130-lacking viruses is completely resistant to neutralization by the elicited anti–V2 apex antibodies. Does this resistance reflect antibody features other than CDRH3? Is this related to the lack of neutralization of N130-expressing viruses? This point warrants more prominent discussion.
4. Lines 143-149: To determine whether the serum antibody neutralizing responses (Ext Fig 1f) following the Q23 and ZM233 immunizations target the V2 apex of Env, EMPEM assays was performed. EMPEM does not report on the neutralizing potential of bound antibodies. That phrase should be corrected. Also, if the intent was to determine whether the neutralizing activities of purified serum IgG was due to apex antibodies, why not use the purified IgG (rather than serum) during EMPEM?
5. At P2, 4 of 6 soluble trimer–immunized NHPs generate anti-base antibodies (Ext Fig. 1g), but so do at least 5 of 6 trimer-liposome–immunized NHPs (Fig. 1f). How are anti-base antibodies arising in the liposome group if the trimer bases are covalently attached and expected to be shielded from B cell recognition by high Env density?
6. Lines 314–316: The authors suggest that their prime-boost strategy is preferable to regimens using boosts designed to progressively increase affinity for maturing antibody intermediates. This conclusion is not obvious. For instance, WITO.33 is not neutralized by 11 of 12 sera (Ext Fig. 1h), suggesting that an Env with intermediate affinities might have been more effective within this schema.
7. Please clarify which viruses in Fig. 1 and Ext Fig. 1 are apex-sensitive and what is meant by “apex-sensitive.”
8. Lines 164-169: The statement that RW020 and CH119 NFL immunizations “broadened” cross-neutralization is not obvious from comparing Fig. 1g and Ext Fig. 1h. Please clarify what is meant by “broadened.”
9. B cell sorting: How many PBMCs were used to sort Env^+ B cells? A table listing the number of sorted B cells, sampling time points, and probes used (Q23, BG505.) and when they were used alone or in combination would be helpful.
10. A strength of the study is the isolation and characterization of many Env^+ B cells across animals. However, lines 178–179 note that B cell analysis focused on NHPs Q9, Q10, and Q12. Why was Q10 included instead of Q8, which showed broader serum breadth (Fig. 1g)? Also, why not isolate B cells from animals Q7 or Q11, whose sera display broader breadth than Q12, for example?
11. Among the four mAbs with solved crystal structures (Ext Fig. 3), Q10M-055 has a non-protruding CDRH3 yet shows greater breadth than Q9M-023, whose CDRH3 protrudes. Does this indicate that CDRH3 protrusion is not the primary determinant of breadth, and that other features (see comment 2) are involved? Are cryo-EM data available for Q10M-055 bound to Env?
12. Abstract lines 37-39: Why emphasize similarity to PG9 when the elicited antibodies do not neutralize N130-bearing viruses, while PG9 does?

Minor

1. Consider introducing “envelope glycoprotein (Env) spike” at line 28 rather than line 31.
2. Line 34-36: consider revising to: “Critically, we isolated monoclonal antibodies (mAbs) from multiple macaques that…”

Referee #3

(Remarks to the Author)

The manuscript titled “HIV Env apex broadly cross-reactive neutralizing antibodies by vaccination” by Javier Guenaga and colleagues reported an improved vaccination scheme of HIV-1 vaccine in NHP model that led to elicitation of cross-neutralizing HIV serum antibody responses. To achieve this, the authors used liposomes for covalent-multivalent display of heterologous HIV Env trimers designed based on near-native NFL-stabilization, they selected Q23.17 strain as priming immunogen using the HIV Env V2-apex antibody RH1 and its inferred germline as hinted from previous studies. And then boosted with Env-liposome using the same liposome technology. This study contributes to the fields of HIV-1 vaccine development with several critical improvements. Most importantly, immune responses from all liposomes immunized NHPs were cross-reactive, and the strategy used here not only triggered the germlines of target V2-apex antibodies (where most of the current publications in the field are at), the somewhat “short 2-year” regimen used here also guided B cells to take the next steps to elicited broad neutralizing antibodies (in all test animals). The cryo-EM structures clearly showed that the vaccine elicited NHP-solution had good mimicry of infection-induced human antibodies, in mode of recognition and at atomic-level, eg, the engagement of C stand of V2 loop. It provides an alternative or improved strategy to the HIV vaccine field.

I have only a few questions:

1. Did the author observe any sulfated Tyr in the CDR H3?
2. For the SHMs developed along the immunization, what are the critical once that essentially enabled the breadth?
3. Based on the analysis of b cell repertoires and the boosting immunogens, possible to deduce which of the boosts are more critical that others? And possible to come out an shortened scheme that may have the same outcome?

Version 2:

Reviewer comments:

Referee #2

(Remarks to the Author)

The authors responded to my queries appropriately and provided additional experimental data to support their conclusions. Their response to my query about why the characterized mAbs are less effective in neutralizing N130 glycan-expressing viruses is logical and the new data provided in the revised manuscript indicating that a small fraction of the characterized mAbs may be able to neutralize N130 glycan expressing viruses is strength.

Referee #3

(Remarks to the Author)
The authors have address all my comments.
Only one suggestion for presentation in Figure 3B, can the HCDR3s be aligned along the "red" motif residues, not using the center justified?

Open Access This Peer Review File is licensed under a Creative Commons Attribution 4.0 International License, which permits use, sharing, adaptation, distribution and reproduction in any medium or format, as long as you give appropriate credit to the original author(s) and the source, provide a link to the Creative Commons license, and indicate if changes were made.
In cases where reviewers are anonymous, credit should be given to 'Anonymous Referee' and the source.
The images or other third party material in this Peer Review File are included in the article's Creative Commons license, unless indicated otherwise in a credit line to the material. If material is not included in the article's Creative Commons license and your intended use is not permitted by statutory regulation or exceeds the permitted use, you will need to obtain permission directly from the copyright holder.
To view a copy of this license, visit https://creativecommons.org/licenses/by/4.0/

Point-by-point Response to Reviewers

- We have responded to all Reviewer comments (in italics below) with our Responses and, where appropriate, modified the revised manuscript, Figures or added new supporting data/experiments accordingly.

Reviewer 1

Comment: *Wyatt and colleagues report an important finding: vaccination of rhesus macaques (RMs) with multivalent, high avidity, tandem Q23 and ZM233 Env trimers bound to liposomes (followed by additional wild-type Env trimer-liposome boosts) induced V2 apex-targeted broadly-neutralizing serum antibodies (bnAbs) in 6 of 6 monkeys. This result – consistent induction of potent V2 apex ns in serum of outbred animals following immunization – has not been previously achieved by any investigative group despite decades of work and represents a watershed moment in HIV-1 vaccine research. The only other studies that approximate this are those showing that extensive immunizations with fusion peptide conjugated to KLH or tetanus toxin followed by Env trimer SOSIP vaccination elicits broadly-reactive but generally low potency antibody responses in small animals and nonhuman primates.*

Response. We thank Reviewer 1 for these very positive comments on our study.

The Wyatt team isolated bnAb mAbs from monkeys with the greatest neutralization breadth and showed that these largely recapitulate the breadth seen in serum. With these mAbs in hand, they could characterize their immunogenetics and structures and confirm the authenticity of the V2 apex targeted responses. Thus, the authors prove for the first time in an outbred animal model that V2 apex bnAb lineages can be efficiently primed by a protein immunogen and relatively easily affinity-matured to acquire neutralization breadth and potency.

The elegance and impact of this manuscript is in its simplicity: take "special Envs" known for their predilection for binding V2 apex bnAbs and bnAb iGL receptors; enhance dramatically their valency and avidity for bnAb precursors; deliver a lot of them (800 ugm) repetitively (q2wks) over an initial 8 week period to a lot of naïve germline target cells (8 separate immunization sites, two in each of four extremities) and immunize five more times over a two-year period . . . and presto: bnAbs!

Response: We agree with Reviewer 1 fully regarding their comments on our paper (although we would note that we do achieve some level of initial cross-neutralization in the serum IgG within 4 trimer immunizations within a year, see Extended Data Fig.2/).

A potentially important inference that the authors draw that may impact HIV-1 immunogen design more generally is that a key to their success was the use of near-native-like Envs for prime and boosts as opposed to heavily-engineered Envs. The authors suggest by using wildtype Envs as immunogens and increasing their avidity by multivalency, they prime naïve germline B-cells with receptors that from the outset recognize and bind near-native Envs. As a consequence, the next steps of affinity maturation need only "polish" or affinity mature modestly evolving bnAb receptors to bind still more avidly to wildtype Envs and develop breadth and potency. The authors point out that this is a very different strategy than that pursued by some

other leading groups in the HIV-1 vaccine field who prime with heavily engineered Envs that bind particular bnAb iGL receptors well and expand them efficiently but which thus far have been unable to develop neutralization breadth and potency . . . probably because the heavily engineered Env immunogens prime naïve germline B cells that have receptors that "look" like bnAb precursors but which at the atomic level are actually quite different. Wyatt and colleagues don't have this problem because they prime with near-native Env trimers.

Response: We agree with Reviewer 1's interpretation of our approach and results. We agree that generally and specifically in at least this case, 'over-engineering' of the priming immunogen to increase affinity, can activate so-called precursors; however, it remains to be seen how feasible it is to evolve such precursors to bind to the native Env on HIV and mediate broad neutralization.

I do have a number of concerns and issues with thi manuscript:

***Comment:** Line 102 – Bold heading reads "Env recognized by a germline apex bnAb UCA." The authors correctly note that Q23 Env binds to the rhesus V2 apex bnAb RHA1 lineage iGL but this is NOT a UCA. Later in the paragraph refer to enhanced binding of the Q23 liposome to the human V2 apex bnAb CH01 and to the "unmutated common ancestor or UCA" of the CH01 lineage antibodies. The terms "UCA" and "unmutated common ancestor" mean something very specific: a phylogenetic inference where the V, D and J genes plus the nontemplated sequences between V and D and D and J have all been precisely inferred from deep sequencing of Bmem Ig sequences. This was NEVER done for the CH01 lineage and could never have been done because clinical samples from the CH01 subject were not available to do so. Instead, the Haynes group made an "RUA" inference (reverted unmutated ancestor), which is similar to an iGL but certainly nothing approaching a true UCA inference (see Liao et al., Immunity 38:176, 2013 and other Haynes papers). Indeed, one can find in the scientific literature the CH01 lineage precursor referred to as "UCA," "RUA," and "iGL. With respect to the CH01 lineage precursor, use of the term "UCA" is sloppy, misleading and incorrect. Wyatt and colleagues should correct this throughout the manuscript and in Fig. 1D.*

Response: We thank Reviewer 1 for their explanation and clarification of the UCA nomenclature. Accordingly, we changed the section title to specified that the germline bNAb is reverted and not a UCA Line 103. Also we changed the annotation of CH01 UCA to "igL CH01" in the revised manuscript in both the Main text (line 126) and Figure 1d.

Line 136 – I suppose one could say that Q23 and ZM233 immunizations were given in "tandem" but this seems like an awkward and potentially confusing description. Why not write "sequential"? Also, the authors should make clear that the monkeys were primed with Q23 and boosted with ZM233 and then boosted again and again with other wildtype Envs.

Response: We believe that the tandem immunization of Q23 followed by ZM233 is clear as described. We wanted to emphasize that from the data we believe that this immunogen duo was critical for efficient 'priming' of the V2-apex antibody response. Since it is following the ZM233 boost we detect autologous neutralization of both viruses and apex densities in all trimer-liposome

immunized subjects. Never-the-less, we changed the word "tandem" for "sequential" in line 136 as suggested. To clarify the complete immunization regimen, we moved up a sentence (line 142-146) that describes the additional boosting trimers following sequential immunizations with the Q23 and ZM233 NFLs (either soluble or covalently arrayed on the trimer-liposomes).

Comment: *Line130-169 – Figs. 1g and Extended Data Fig 1h, and the text describing these results, are difficult to follow because the viruses in the left-most columns are sometimes the same between the figures, sometimes different, and oftentimes not in the same order top to bottom. Furthermore, the figures are used to highlight the "breadth" of the serum IgG responses but some of these viruses are identical to the immunogens these monkeys received: thus, such responses are autologous, not heterologous. It would be more clear if the authors were to separate spatially in the figures responses to autologous Envs from responses to heterologous Envs and indicate which is which. It would also be helpful if Figs. 1g and Extended Data Fig 1h contained an identical set of heterologous Envs in the same order top to bottom, and it would be even better if the list of viruses was longer and included all of the viruses shown in Fig. 2C at a minimum.*

In other sections of the text, the same confusing situation arises when the authors show breadth and potency of bnAb mAbs, where for example, in Fig. 2c some of the viruses are autologous to the immunogens, others are heterologous, and the order top to bottom doesn't resemble Figs. 1g or Extended Data Fig 1h. What makes this even more difficult to follow is then the authors indicate that some viruses contain N130 and some don't and it is not always clear from the figures or the legends which is which. It might be helpful as an Extended Data figure to have a single table where every virus that was tested for neutralization sensitivity to either serum IgG or mAbs is listed top to bottom; next to that an indication if it contains N130 or not; next to that if it is part of the immunization sequence or not, and next to that the neut results for both polyclonal IgG and mAb. (I don't feel strongly about this latter suggestion if the authors can otherwise address the lack of clarity.)

Response: We thank the Reviewer for these insightful comments and acknowledge that we could improve our paper by a clearer and more organized listing of the HIV strains in the neutralization Tables. Accordingly, we reorganized all neutralization data Tables by HIV-1 strain clade and by numerical/alphabetical order within a specific clade. These modifications should facilitate ease of cross-comparison by the reader. Additionally, we colored the viruses representing Env sequences used in the NFL trimer immunogen series in blue font for ease of identification. Furthermore, we added a new main Figure (new Figure 2) to extend the trimer-liposome group serum IgG neutralization analysis to include the entire panel used for the monoclonals, a total of 67 viruses. We added a new extended data figure (Extended Data Fig. 2a) to show longitudinal neutralization data (P4, P5 and P6) of serum IgG for both NHP groups with a sentinel viral panel. We believe that the additional data and improved presentation of the neutralization panels greatly increase the clarity of the results.

Comment: *Line 171-213 – I found this text and the corresponding Fig. 2a and 2b confusing. The authors state that they recovered 103, 159 and 393 paired H and L chains from monkeys Q9, Q10 and Q12, and these grouped into 70, 106 and 203 clonal lineages. To me, a clonal lineage is defined as a lineage of B*

cells that have evolved from a single germline UCA B cell that was primed and then expanded. If that is what the authors mean, they should say so. Then, they say that they analyzed these lineages and selected those with D3-15 genes and CDRH3s longer than 20aa and a negatively-charged core motif, leading to the identification of 16, 30 and 58 HC and LC pairs, respectively, with these features. Am I correct to assume this means that from Q9, they found 16 expanded bnAb-like lineages, from Q10 they found 30, and from Q12 they found 58. So, for example, using PBMC samples collected 1-2 years post-initial prime, they found 58 distinct bnAb-like lineages? If so, were these primed by Q23, ZM233, WITO or one of the other three immunogens? Finally the authors say they selected 45 HC and LC pairs from this total of 104 to clone and express as mAbs and to assess their neutralization properties . . . leading to the identification of 9 bnAb mAbs corresponding to 5 discreet lineages shown in Fig. 2b. If all this is the case, it would be helpful to the reader to have this spelled out more clearly and for the authors to offer some perspective on how these 5 discreet bnAb lineages relate to the 104 bnAb-like lineages described above. In essence, what I am asking the authors is what is the priming efficiency of their Q23 liposome? How many V2 apex bnAb-like precursors were primed and expanded after the initial prime and boost and how many of these affinity matured with further immunizations to acquire breadth and potency?

Response: We thank the Reviewer for their careful reading of the initial manuscript. We realize that the original wording may have caused confusion regarding the distinction between clonal lineages versus heavy and light chain (HC-LC) pairs, but the Reviewer has interpreted the section correctly. During the revision of the manuscript, we expanded our analysis by including additional antibodies from NHP Q7 and Q9, resulting in larger numbers of paired HC-LC sequences and clonal lineages than reported in the original submission. We updated the corresponding Figures and Tables (now Figure 3a and Figure 3b and Extended Data Table 1b as we have added a new main Figure 2 and a new Table (Extended Data Table 1a) detailing sort statistics for each animal).

We agree that a clonal lineage is defined as sequences that originate from a single activated naïve B cell, which, following proliferation and recruitment into a germinal center (GC) reaction, gives rise to a lineage of cells with BCRs that undergo SHM and selection. We use the same definition of antibody clonal relatedness as is common in the field, i.e. HC-LC sequences should use the same V and J allele, same CDR3 length and high sequence identity across the HCDR3 (80% or higher identity at a nucleotide level; allowing for some SHM).

From the B cell sorts described in the revised paper, now also including Q7 and additional data from Q9, we identified 208, 217, 159 and 393, paired HC and LC sequences from animal Q7, Q9, Q10 and Q12 that grouped into 111, 117, 106 and 203 clonal lineages, respectively. Among these lineages, 13 from Q7 (34 HC-LC pairs), 4 from Q9 (38 pairs), 12 from Q10 (30 pairs) and 17 from Q12 (58 pairs) met the criteria we selected for mAbs isolation to facilitate identification of apex-targeting bNAbs: usage of IGHD3-15 and HCDR3s of at least 20 aa in length.

A large set of these HC-LC pairs was expressed as mAbs of which several displayed cross-neutralizing activities in a sentinel panel detailed in Extended Data Fig. 3a. The best 11 antibodies were selected for further analysis. These 11 antibodies belong to six distinct clonal lineages.

We would like to point out that we likely did not saturate the analysis as we believe that additional sorts could yield additional lineages that target the apex or other neutralizing epitopes. We will pursue a deeper analysis of the repertoires in these animals in a follow-up study. We would also like to point out that in response to Reviewer 2 (below), we added a table summarizing the number of PBMCs used to isolate Env-binding B cells, the number of sorted B cells, sampling time points and probes used (Extended Data Table 1a).

We believe that both the Q23 and ZM233 NFL were likely responsible for the generation of V2-apex targeting lineages early in the immunization scheme as EMPEM data (Figure 1f) detected Fab densities in all trimer-liposome immunized NHPs. Additionally, robust neutralization of Q23.17 and ZM233M.6 HIV-1 strains at P2 suggests that both NFLs contributed to the generation of these lineages. The ontogeny of the lineages in not within scope of this study will be addressed further in a follow-up analysis by performing deep BCR repertoire sequencing and lineage tracing.

We would also like to note that we did not include all mAbs that displayed neutralizing activity in the current paper. Instead, we focused our analysis on the mAbs with the broadest neutralizing activity to share the results of these exceptional vaccine-induced antibodies in a timely manner.

We have now revised the manuscript to clearly distinguish between the paired HC-LC sequences and the clonal lineages (lines 196-202).

Reviewer 2

Key result: The study demonstrates effective immuno-focusing on the V2 apex of the HIV Env in NHPs, leading to the elicitation of tier 2 HIV-1 neutralizing antibodies targeting this region. Validity: No apparent flaws.

Originality/significance: The results are highly significant.

The findings are significant. The central concept is compelling, the experiments were conducted in a relevant animal model, and the conclusions are well supported by the experimental data. The elicited breadth of neutralization against heterologous tier 2 viruses that is focused on the V2 Apex of Env, is impressive, but is limited to only a subset of viruses with Envs lacking the N130 glycans. The underlying reasons for the neutralization resistance by a large fraction of N130-expressing viruses are not discussed. Although the authors correctly note that the CDRH3 regions of the elicited anti-Apex antibodies share features with known anti-V2 Apex broadly neutralizing antibodies, the underlying mechanistic reasons for their failure to neutralize N130-expressing viruses but also N130-lacking viruses, remain unclear and are not addressed.

Response: We agree with Reviewer 2 that these are interesting and important questions, and, like the reviewer, we wonder about this issue of the glycan at position N130 which is present in approximately 50% of HIV-1 strains. The cryoEM structures presented here (complexes of N130 lacking NFL Env trimers and the NHP antibodies) offer some clarity. The light chain CDR1 loop of the antibodies is located at a distance that leaves little clearance for a complex glycan potentially resulting in a clash if the glycan were present. We now have evidence that some of these antibodies

(Q9M-023 and Q9M-246) can neutralize an N130-expressing Q23.17 virus suggesting that this potential clash can be obverted or overcome in some cases (Extended Data Fig. 3b). The Env-probes used for sorting B cells were lacking the N130 glycan and thus we sorted antibodies that generally prefer that context.

Regarding the neutralization of N130-lacking viruses, we pinpoint the divergence of the V2 C strand Env sequence in the section "HIV Env V2 epitope mapping analysis" as one potential contributing factor. In that regard, we have now added analysis of an additional mAb from NHP Q7 (Q7M-675) that can neutralize a divergent C strand virus (WITO.33) (Figure 4d). This antibody neutralizes the WITO.33 I169R variant more potently, suggesting that the C strand divergence impacts the neutralization sensitivity of the antibody. Both Q7 and Q8 sera IgG (P6) display the exact same neutralization sensitivity pattern as Q7M-675, strengthening the argument of the C strand sequence divergence as an issue to neutralization sensitivity of N130 glycan-lacking viruses.

Comment: *1. Lines 86-92: After the first two immunizations, autologous neutralizing antibodies were detected in both liposome- Env and soluble Env–immunized animals, yet only apex-directed antibodies in the liposome group are discussed here. They should mention that such responses were also recorded in the soluble Env immunized animals, but at lower frequences.*

Response: The Apex-directed antibody responses detected by EMPEM in both groups are mentioned in lines 146-153 and shown in Figure 1f and Extended Data Fig. 1g. We also added a new EMPEM magnitude analysis and a corresponding Figure panel (Fig. 1g) that denotes all epitope responses detected by EMPEM in both groups early in the immunization regimen (P2, that is following the 2nd trimer immunogen, ZM233). These data show that apex responses were detected in four NHPs immunized with soluble trimers at bleed P2 following the sequential immunizations with the Q23 and ZM233 NFLs (and correlated with more modest autologous neutralization than elicited in the trimer-liposome animals). In these NHPs, the serum IgG autologous and cross-neutralization was detected in all animals immunized sequentially with the NFL trimer-liposomes (Extended Data Fig. 2a).

Comment: *2. The IgL form of RHA1, which carries the same CDRH3 as the mature antibody, shows minimal or no binding to Q23NFL and ZM233 NFL, while the mature antibody binds them with KDs near 10^{-10} (Fig. 1b). This suggests that the CDRH3 alone is insufficient for broad neutralization. Which additional features of V2 apex-directed bnAbs contribute to Env engagement and breadth? Do the elicited NHP antibodies lack these features? Understanding this would clearly inform improved immunogen design.*

Response: This is an interesting point that is brought up by Reviewer 2. The Q23 NFL displays micromolar affinity for the iGL RHA1 antibody and as the Reviewer mentions this affinity alone is insufficient to mediate neutralization of the Q23.17 virus. The human bnAbs, like PG9, and the macaque bNAbs, presented in this study, interact with Env in a very similar way. They make strong electrostatic interactions with the Env trimer strand C of V2 region and they make extensive interactions with two of the N160 glycans at the spike apex. We show in Figure 4a and 4b and Extended Data Fig. 4a, how these glycans interact in a positive manner with specific monoclonal

antibodies. Elimination of the N160 glycan sequon in the context of either the Q23.17 or BG505 viruses results in most cases as a marked to partial decrease in neutralizing potency. These data support the role of antibody:glycan interaction as a major contributor to the antibody neutralizing activity. It is our opinion that antibody interactions with glycan are likely essential for the development of neutralization breadth and potency.

Comment: *3. Ext Fig. 2: A substantial fraction of N130-lacking viruses is completely resistant to neutralization by the elicited anti–V2 apex antibodies. Does this resistance reflect antibody features other than CDRH3? Is this related to the lack of neutralization of N130-expressing viruses? This point warrants more prominent discussion.*

Response: We believe that the neutralization resistance of N130-lacking viruses is due to the variability of the strand C of V2 as we describe in the section "HIV Env V2 epitope mapping analysis" and discussed earlier in this Response letter. Figure 4c shows how Q12BBM-069, our most potent and broad macaque antibody, can neutralize most of the viruses that include strand C sequences with high degree of similarity to that of the immunogens used in the vaccine regimen ($_{164}$ELRDKKQKV$_{172}$ consensus). Viruses on the right column of Figure 5c that are not neutralized possess divergent strand C sequences. We attribute the resistance phenotype of viruses lacking-N130 to the C strand variability, especially when a positive charge is substituted. And this extends to the N130-expressing viruses. However, there may be other contributing factors, such as variability in V1 or/and glycosylation in V2b that can interfere with antibody epitope recognition. We extended the Discussion of this topic as suggested by the Reviewer (lines 355-363).

Comment: *4. Lines 143-149: To determine whether the serum antibody neutralizing responses (Ext Fig 1f) following the Q23 and ZM233 immunizations target the V2 apex of Env, EMPEM assays was performed. EMPEM does not report on the neutralizing potential of bound antibodies. That phrase should be corrected. Also, if the intent was to determine whether the neutralizing activities of purified serum IgG was due to apex antibodies, why not use the purified IgG (rather than serum) during EMPEM?*

Response: We agree with the reviewer that EMPEM does not necessarily inform fully the neutralization capacity of the PolyFabs visualized. We have now rephrased the statement to say "To determine whether the elicited antibody responses were targeting the HIV Env apex, we performed EMPEM…". Lines 146-150.

EMPEM analysis in this study does use the purified serum IgG. The IgG fraction is isolated by protein A purification from 1 mL of serum. The IgG is then enzymatically processed to generate the polyFabs used in EMPEM analysis in complex with the well-ordered and stable NFL trimers.

Comment: *5. At P2, 4 of 6 soluble trimer–immunized NHPs generate anti-base antibodies (Ext Fig. 1g), but so do at least 5 of 6 trimer-liposome–immunized NHPs (Fig. 1f). How are anti-base antibodies arising in the liposome group if the trimer bases are covalently attached and expected to be shielded from B cell recognition by high Env density?*

Response: We have now performed a new analysis that measures the frequency of epitope-specific antibody responses derived from EMPEM data. We added a new Figure panel (Figure 1g) and revised the manuscript accordingly (lines 153-155) displaying graphically and quantitatively the results of the new analysis. The frequency of trimer-base targeting antibodies is significantly lower in the liposomal group samples. The base responses do still exist but at a much lower frequency than in the soluble trimer samples. That this occurs despite covalent tethering of the NFL trimers to the liposome might be a result of ultimate degradation of the liposome *in vivo*.

6. Lines 314–316: The authors suggest that their prime-boost strategy is preferable to regimens using boosts designed to progressively increase affinity for maturing antibody intermediates. This conclusion is not obvious. For instance, WITO.33 is not neutralized by 11 of 12 sera (Ext Fig. 1h), suggesting that an Env with intermediate affinities might have been more effective within this schema.

Response: We agree with the Reviewer that sequential immunogens could be selected based on affinity increments of antibodies elicited by prior immunization. However, in this study, we selected boosting immunogens based on recognition by known infection-elicited V2-apex bNAbs.

We would like to comment on the statement noted by Reviewer 2 (lines 342-348) that it is our belief that priming with a natural Env sequence might be a desirable strategy to artificially enhance affinity to a B cell bnAb precursor by extensively modifying the Env epitope with unnatural (non-native) residues. Extensive unnatural Env alterations will require further boosts to get the antibodies to recognize Env while in the case of our strategy, using natural Env residues, the recognition happens with priming.

Comment*: 7. Please clarify which viruses in Fig. 1 and Ext Fig. 1 are and what is meant by "apex-sensitive."*

Response: We agree that there is not a rigorous definition for "apex-sensitive" viruses or Envs. We define "apex-sensitive" as HIV-1 strains that display greater sensitivity of neutralization to known V2-apex bNAbs than do the majority of clinical isolates. Most of these sequences have 3 to 4 positively charged amino acids in the C-strand of V2 and often lack the N130 glycan. We have removed this wording from the manuscript to avoid ambiguity.

Comment*: 8. Lines 164-169: The statement that RW020 and CH119 NFL immunizations "broadened" cross-neutralization is not obvious from comparing Fig. 1g and Ext Fig. 1h. Please clarify what is meant by "broadened."*

Response: To clarify this comment, we added a sentence in the revised manuscript (line173-184). Specifically, NHPs Q7, Q8, Q9, Q10 and Q11 gained neutralization activity (from 1 to 6 viruses) from the activity measured at the P4 time point as compared to P6 in this limited longitudinal panel (Extended Data Fig. 2b).

Comment: *9. B cell sorting: How many PBMCs were used to sort Env⁺ B cells? A table listing the number of sorted B cells, sampling time points, and probes used (Q23, BG505.) and when they were used alone or in combination would be helpful.*

Response: We thank the Reviewer for this suggestion. We have added the requested sorting details for each animal in a new Table, now included as an Extended Data Table 1a. This Table lists the sampling time points, the probes used, the number of stained PBMCs, the number of sorted B cells, the resulting productive HC-LC pairs, and the mAbs obtained from each sort. The mAbs belonging to the same clonal lineage are marked with identical symbols.

Comment: *10. A strength of the study is the isolation and characterization of many Env⁺ B cells across animals. However, lines 178–179 note that B cell analysis focused on NHPs Q9, Q10, and Q12. Why was Q10 included instead of Q8, which showed broader serum breadth (Fig. 1g)? Also, why not isolate B cells from animals Q7 or Q11, whose sera display broader breadth than Q12, for example?*

Response: We thank the Reviewer for this question. During the revision, we expanded our analysis by including additional mAbs from NHP Q7 and Q9. The corresponding Figures and Tables (Figure 3a, b, c, Extended Data Figure 3a, b) have been updated accordingly. The original Extended Data Table 1, which summarizes the genetic features of the expressed mAbs, has been expanded and renumbered as Extended Data Table 1b. We now have mAbs from 4 animals out of 6 immunized with the trimer-liposomes. Q12 and Q9 were judged a priority since their serum IgGs displayed the most potent and broad neutralization at the P4/P5 timepoints, respectively.

11. Among the four mAbs with solved crystal structures (Ext Fig. 3), Q10M-055 has a non-protruding CDRH3 yet shows greater breadth than Q9M-023, whose CDRH3 protrudes. Does this indicate that CDRH3 protrusion is not the primary determinant of breadth, and that other features (see comment 2) are involved? Are cryo-EM data available for Q10M-055 bound to Env?

Response: This is an interesting point raised by this Reviewer. We do have a cryoEM structure of Q10M-055 in complex with the Q23 NFL which is featured in Extended Data Figure 5. As the reviewer points out, Q9M-023 does have a slightly longer HCDR3 than Q10M-055 (24 aa vs 22 aa, respectively, Figure 3b and Extended Data Figure 5b). However, as we discussed in the manuscript (Lines 266-268) Q10M-055 possesses sulfated Tyrosine (Y100b) at the tip of the loop that extends its reach to match that of the other antibodies with longer HCDR3s (Extended Data Figure 5a, right panel).

We would like to clarify that the antibody "alone" crystal structure shown in Extended figure 4a does not show a protruding HCDR3 because there was no electron density resulting from the crystal X-ray diffraction suggesting a more flexible loop than the other antibodies. Perhaps, the HCDR3 flexibility might be a contributing factor to its broad activity as the Reviewer points out.

12. Abstract lines 37-39: Why emphasize similarity to PG9 when the elicited antibodies do not neutralize N130-bearing viruses, while PG9 does?

Response: We do feel strongly about this comparison based on structural and functional feature similarities. PG9 is indeed a superior antibody than the macaque antibodies featured in this analysis. The NHP antibodies presented here share a high degree of structural similarity with PG9 more than they do with any other V2 apex bnAb elicited by infection. The disposition of the heavy and light chains, the insertion of the HCDR3 between the N160 glycans, the interaction with the glycans and the Env V2 C-strand are structural similarities that make this comparison valid (Figure 4).

As the Reviewer notes, the N130 glycan poses no obstacle for PG9 neutralization while the macaque antibodies presented here cannot generally overcome the N130-glycan obstacle. However, in the revised manuscript we have included new data that shows that a few of the macaque bNAbs can neutralize the Q23.17 H130N virus when the N130 glycan sequon is restored, suggesting that perhaps with further maturation the breadth of neutralization could be expanded by modifications of the immunization trimers and/or regimen. In addition, as shown in the new Figure 2 there is neutralization detected of several N130-glycan-containing viruses although we don't yet know for certain this activity is from apex-directed antibodies. Further, our immunogens all lacked the N130 glycan as did our sorting probes, so there may be antibodies yet to be identified that can neutralize N130-containing viruses.

Minor

Comment: 1. Consider introducing "envelope glycoprotein (Env) spike" at line 28 rather than line 31.

Response: We introduced the change requested. We thank the Reviewer for the suggestion.

Comment: 2. Line 34-36: consider revising to: "Critically, we isolated monoclonal antibodies (mAbs) from multiple macaques that…"

Response: We thank the reviewer for this suggestion and have revised the wording accordingly.

<u>Reviewer 3</u>

Comment: *1. Did the author observe any sulfated Tyr in the CDR H3?*

Response: We thank Reviewer 3 for this question. Yes, Q10M-055 possesses a sulfated tyrosine (Y100b) at the tip of the HCDR3 that interacts with the R169 side chain and the asparagine of the N160 glycan. This interaction stabilizes the HCDR3 loop that is partially disordered in the unliganded crystal structure. This sulfated tyrosine-Env interaction occurs with the most distal Env protomer, increasing the antibody's reach despite its slightly shorter HCDR3 (Extended Data Fig.

5a, right panel). We made this panel larger, and we also annotated the presence of the sulfated tyrosine in the Figure legend.

Comment: *2. For the SHMs developed along the immunization, what are the critical once that essentially enabled the breadth?*

Response: We agree that this is an interesting question that merits additional analysis that is outside the scope of this first paper. In the future, we will perform both SHM reversion mutagenesis in specific apex bNAbs coupled with lineage tracing analyses of specific mAbs isolated from longitudinal samples that should provide clearer insights regarding which SHM changes are most critical to evolve neutralization breadth. At this juncture, one possibility is that the SHM results in increased affinity for the highly conserved apex glycans at residue N160, which we see stabilized in several of the mAb:trimer high resolution cryoEM structures. It is possible that the higher affinity for antibody with the conserved glycans contributes to the broad neutralizing activity detected in of several monoclonal antibodies isolated at later timepoints of the study.

Comment*: 3. Based on the analysis of b cell repertoires and the boosting immunogens, possible to deduce which of the boosts are more critical that others? And possible to come out an shortened scheme that may have the same outcome?*

Response: We thank Reviewer 3 for this question. The important results presented here provide a positive control 'foundation' for future optimization vaccine studies that allow us to alter single variables in the initial vaccine regimen to potentially reduce the number of trimer boosts required to elicit apex-directed broad neutralization. Based on the initial detection of cross-neutralization we believe that at least 3 of the first 4 NFL immunogens are likely critical to the positive outcome presented here. We would speculate that perhaps the WITO NFL might be omitted for future studies as that trimer possesses a more divergent C-strand than does the 4th 1428 NFL…and, as well, this trimer did not efficiently elicit autologous neutralization in most animals as did the other 3 early immunogens.

The last two inoculations could perhaps be better selected using the monoclonals we have now isolated in this study. A shortened vaccine scheme is in our plans moving forward by reducing the number of boosts. Future single-variably experiments will be designed to address these questions to better allow translation of these results in NHPs to testing of optimal vaccine candidates/regimens in humans.

Point-by-point response to referees' comments

Below are our responses, drafted in blue font, to concerns raised by the referees in the last revision.

Referee #2

"The authors responded to my queries appropriately and provided additional experimental data to support their conclusions. Their response to my query about why the characterized mAbs are less effective in neutralizing N130 glycan-expressing viruses is logical and the new data provided in the revised manuscript indicating that a small fraction of the characterized mAbs may be able to neutralize N130 glycan expressing viruses is strength."

Response:

We thank referee #2 for the positive comments.

Referee #3

"The authors have address all my comments.
Only one suggestion for presentation in Figure 3B, can the HCDR3s be aligned along the "red" motif residues, not using the center justified?"

Response:

We have now aligned the HCDR3 sequences featured in Figure 3b along the negatively charged "red" residues and we also have changed the font to Courier to conform to the journal formatting guidelines of amino acid sequences.
